# Mapping Recombination Rate on the Autosomal Chromosomes Based on the Persistency of Linkage Disequilibrium Phase Among Autochthonous Beef Cattle Populations in Spain

**DOI:** 10.3389/fgene.2019.01170

**Published:** 2019-11-20

**Authors:** Elena Flavia Mouresan, Aldemar González-Rodríguez, Jhon Jacobo Cañas-Álvarez, Sebastián Munilla, Juan Altarriba, Clara Díaz, Jesús A. Baró, Antonio Molina, Pascual Lopez-Buesa, Jesús Piedrafita, Luis Varona

**Affiliations:** ^1^Departamento de Anatomía, Embriología y Genética Animal, Universidad de Zaragoza, Zaragoza, Spain; ^2^Departament de Ciència Animal i dels Aliments, Universitat Autònoma de Barcelona, Barcelona, Spain; ^3^Departamento de Producción Animal, Facultad de Agronomía, Universidad de Buenos Aires, CONICET, Buenos Aires, Argentina; ^4^Departamento de Mejora Genética Animal, Instituto Nacional de Investigación y Tecnología Agraria y Alimentaria (INIA), Madrid, Spain; ^5^Instituto Agroalimentario de Aragón (IA2), Zaragoza, Spain; ^6^Departamento de Ciencias Agroforestales, Universidad de Valladolid, Valladolid, Spain; ^7^Departamento de Genética, Universidad de Córdoba, Córdoba, Spain; ^8^Departamento de Producción Animal y Ciencia de los Alimentos, Universidad de Zaragoza, Zaragoza, Spain

**Keywords:** recombination rate, linkage disequilibrium, beef cattle, multiple populations, gene ontology

## Abstract

In organisms with sexual reproduction, genetic diversity, and genome evolution are governed by meiotic recombination caused by crossing-over, which is known to vary within the genome. In this study, we propose a simple method to estimate the recombination rate that makes use of the persistency of linkage disequilibrium (LD) phase among closely related populations. The biological material comprised 171 triplets (sire/dam/offspring) from seven populations of autochthonous beef cattle in Spain (Asturiana de los Valles, Avileña-Negra Ibérica, Bruna dels Pirineus, Morucha, Pirenaica, Retinta, and Rubia Gallega), which were genotyped for 777,962 SNPs with the BovineHD BeadChip. After standard quality filtering, we reconstructed the haplotype phases in the parental individuals and calculated the LD by the correlation -*r*- between each pair of markers that had a genetic distance < 1 Mb. Subsequently, these correlations were used to calculate the persistency of LD phase between each pair of populations along the autosomal genome. Therefore, the distribution of the recombination rate along the genome can be inferred since the effect of the number of generations of divergence should be equivalent throughout the genome. In our study, the recombination rate was highest in the largest chromosomes and at the distal portion of the chromosomes. In addition, the persistency of LD phase was highly heterogeneous throughout the genome, with a ratio of 25.4 times between the estimates of the recombination rates from the genomic regions that had the highest (BTA18-7.1 Mb) and the lowest (BTA12-42.4 Mb) estimates. Finally, an overrepresentation enrichment analysis (ORA) showed differences in the enriched gene ontology (GO) terms between the genes located in the genomic regions with estimates of the recombination rate over (or below) the 95^th^ (or 5^th^) percentile throughout the autosomal genome.

## Introduction

Recombination caused by crossing-over during meiosis play a crucial role in the genetic diversity and the genome evolution of organisms with sexual reproduction (Arnheim et al., 2007). It creates new genetic variation by generating novel combinations of grand-paternal and grand-maternal genetic information, and it helps to remove deleterious mutations that might otherwise accumulate (Tiemann-Boege et al., 2017).

In most studies of genome-wide association or genomic selection, the distribution of crossing-over events had been considered uniform, although there is strong evidence that recombination rate is heterogeneous along the genome (Myers et al., 2005; Stapley et al., 2017). In general, recombination is higher in the regions of the telomeres and smaller near the centromere (Coop and Przeworski, 2007; Ma et al., 2015). Due to recombination, the genome is organized into haplotype blocks of varying lengths, as described in humans (Gabriel et al., 2002) and other species as rat and mouse (Guryev et al., 2006) and cattle (Mokry et al., 2014). The reason of this structure is the presence of small genomic regions that have a higher rate of recombination, known as recombination hotspots (Paigen and Petkov, 2010).

In addition, patterns of the recombination rate throughout the genome vary among species, populations, or even within individuals in different environments (Stapley et al., 2017). The evolution of the distribution of the recombination rate along the genome is an active research field (Dapper and Payseur, 2017). In general, it differs according to the genomic scale in which the recombination rate is measured (Smukowski and Noor, 2011). In a very fine scale (few kb), a rapid divergence of the recombination rate between mammal populations is observed (Auton et al., 2012; Stevison et al., 2016), whereas greater correlations are observed between closely related populations when they are calculated through larger chromosomal segments (Smukowski and Noor, 2011; Shen et al., 2018).

Traditionally, the distribution of the crossing-overs or recombination events within the genome has been studied by counting the number of chiasmata during meiosis (Hulten et al., 1982) or from linkage maps created from a limited number of genetic markers or phenotypes (Sturtevant, 1913). In recent years, high-throughput sequencing and genotyping technologies have provided a valuable new tool for measuring recombination rates with two main group of methods. First, estimates of recombination rates are based on observations of recombination events in large pedigrees between pairs of parent-offspring genotypes (Kong et al., 2010; Ma et al., 2015; Shen et al., 2018) or in sperm typing (Sarbajna et al., 2012) and require genotypic information from a large number of families or sperm cells. Second, other methods are based on the identification of local patterns in linkage disequilibrium (LD) with coalescent methods (McVean et al., 2002; Li and Stephens, 2003; Wall and Stevison, 2016), which estimate the background recombination rate *ρ* *_w_*=4*N* *_w_* *c* *_w_* −, where *N* *_w_* and *c* *_w_* are the indistinguishable effective population size and recombination rate for a specific window of the genome, respectively. The main limitation of the last approach is that the effective population size can vary dramatically over time. In fact, the decay of LD has been used to estimate past population history in humans (Hayes et al., 2003; Tenesa et al., 2007; Park, 2012) and livestock populations (Hayes et al., 2003; De Roos et al., 2008; Xu et al., 2019).

In addition, stratification of the population can severely distort the estimates of recombination rates because subdivisions of the population have a strong effect on LD estimates (Hinrichs et al., 2009). After reproductive isolation, the structure of LD tends to differ between subpopulations and the similarity (or persistency) of those LD patterns depends on the number of generations of divergence and the recombination rate plus other evolutionary events such as admixture or variations on the effective size of the populations (Hill and Robertson, 1968). For this reason, if genotypic information is available for closely related populations, measures of genome-wide persistency of LD phase among populations throughout the genome can be used to infer the distribution of recombination rate. The rationale of this approach is that genetic drift, admixture, or variations of the effective size should affect the entire genome with similar intensity and the heterogeneity of the persistency of LD phase is linked to variations on the recombination rate.

Therefore, the objective of this study was to develop a procedure to infer the distribution of the recombination rate from the persistency of LD phase among closely related populations and to apply it to genotypic data from seven beef cattle populations in Spain.

## Materials and Methods

The genomic data comprised the *BovineHD Genotyping Beadchip (777,962 SNPs, Illumina)* genotypes from 171 non-related triplets of sire, dam, and one offspring from seven breeds, being 25 *Asturiana de los Valles* (AV), 24 *Avileña - Negra Ibérica* (ANI), 25 *Bruna dels Pirineus* (BP), 25 *Morucha* (Mo), 24 *Pirenaica* (Pi), 24 *Retinta* (Re), and 24 *Rubia Gallega* (RG) triplets. This dataset has been used to analyze genetic differentiation (Cañas-Álvarez et al., 2015; Cañas-Álvarez et al., 2016; González-Rodríguez et al., 2017), signatures of selection (González-Rodríguez et al., 2016), and haplotype diversity (Mouresan et al., 2017). These breeds represent 72% of the total census of local beef breeds in Spain (Ministerio de Medio Ambiente y Medio Rural y Marino, 2010) and their production systems are extensive or semi-extensive. The populations are reared in mountainous regions near the Pyrenees (*Pirenaica* and *Bruna dels Pirineus*) in the humid regions in northwestern Spain (*Rubia Gallega* and *Asturiana de los Valles*) or in pastures in semi-arid zones of the west and southwest of Spain (*Retinta*, *Avileña Negra-Ibérica*, and *Morucha*). The breeds differ in production and carcass traits (Piedrafita et al., 2003) and in their meat quality (Gil et al., 2001).

The triplets were sampled by the breeders associations with the aim of capturing most of the genetic variability of each population. We used an *ad-hoc* procedure that started with one triplet and incorporated the new ones by minimizing the total coancestry between them. The SNP filtering process included the following: 1) Mendelian error < 0.05, 2) SNP and individual call rates > 95%, and 3) Minor allele frequency (MAF) > 0.05 in pairs of populations. Only the SNPs that were located on autosomal chromosomes were retained. The filtering process yielded approximately 550,000 segregating markers for each pair of populations (see **Table 1**).

**Table 1:** Number of co-segregating SNP markers between all possible pairs of seven beef cattle populations in Spain [Asturiana de los Valles (AV), Avileña - Negra Ibérica (ANI), Bruna dels Pirineus (BP), Morucha (Mo), Pirenaica (P), Retinta (Re), Rubia Gallega (RG)].

Pairs of populations	N° SNP markers	Pairs of populations	N° SNP markers
**AV-ANI**	555,373	**BP-Mo**	543,305
**AV-BP**	557,588	**BP-Pi**	534,336
**AV-Mo**	555,769	**BP-Re**	535,997
**AV-Pi**	540,390	**BP-RG**	544,350
**AV-Re**	547,893	**Mo-Pi**	529,281
**AV-RG**	553,868	**Mo-Re**	541,225
**ANI-BP**	538,327	**Mo-RG**	542,682
**ANI-Mo**	545,324	**Pi-Re**	522,670
**ANI-Pi**	524,630	**Pi-RG**	529,577
**ANI-Re**	536,595	**Re-RG**	535,677
**ANI-RG**	537,882		

The genomic information of the triplets was used to reconstruct the parental haplotypes with the TRIO option of the *BEAGLE* software (Browning and Browning, 2007), which were used to calculate the LD in each population and between each pair of markers (i.e. with alleles A and a, and B and b, respectively) that had a genomic distance < 1 Mb. LD was estimated as a correlation –*r*-, as follows:

$$r=\frac{D}{\sqrt{p_{A}p_{a}p_{B}p_{b}}}$$

where *D* = *P* *_AB_* *P* *_ab_* *P* *_Ab_* *P* *_aB_* (Falconer and Mackay, 1996), *P* *_AB_* *, P* *_ab_*, *P* *_Ab_* and *P* *_ab_* were the haplotype frequencies, and *P* *_A_*, *P* *_a_*, *P* *_B_*, and *P* *_b_* were the allelic frequencies.

To estimate the persistency of LD phase between pairs of populations, the Pearson correlations between LD estimates in each bin of 20 kb (0–20, 20–40, 40–60, 960–980, 980–1,000) within a 1 Mb window were calculated for each pair of populations. We obtained 50 correlation estimates (one per bin) between the LD estimates of each breed pair per window.

Under the assumption of constant variance of *r* (or effective population size) in both populations, the expectation of the correlation between LD estimates from a pair of SNP markers is e^-2cT^ (Hill and Robertson, 1968; De Roos et al., 2008), where *c* is the recombination rate between the markers and T is the number of generations of divergence between populations. Initially, it was assumed that the recombination rate was 1.25 cM per Mb (Arias et al., 2009). The regression of the natural logarithm of the correlations on the genomic distance was calculated and the slope was equated to -2cT to estimate T between each pair of populations.

Once the numbers of generations of divergence (T) were estimated from all available SNP markers, they were assumed as known and replaced by their estimates. Subsequently, the same expression (-2cT) was used to estimate *c*, although the analysis was restricted only to the SNP markers within 1 Mb, which were centered every 0.1 Mb along the autosomal genome in sliding windows. Therefore, 25,098 estimates of c were calculated for each of the 21 population pairs.

Afterwards, the presence of a common pattern for the distribution of the recombination rate was checked calculating the correlation between the estimates from all population pairs. Next, the estimates of *c* were averaged by chromosome, relative position within the chromosome, and location within the genome to infer the distribution of the recombination rate (or the persistency of LD phase) throughout the bovine autosomal genome. The degree of inequality of the recombination rate along the autosomal genome was measured with the Gini index –G- (Ceriani and Verme, 2012) as:

$$G=\frac{\sum_{i=1}^{ng}\sum_{j=1}^{ng}\left|c_{i}-c_{j}\right|}{2ng^{2}\bar{c}}$$

Where *c* *_i_* and *c* *_j_* were the average estimates of the recombination rate for the 21 population pairs ad for the *ith* and *jth* genomic regions, *ng* was the total number of genomic regions (25,098) defined along the autosomal genome, and $\bar{c}$ was the average of the 25,098 estimates of the recombination rate.

Finally, the 5^th^ and 95^th^ percentiles of the average recombination rates between pairs of populations in 0.1 Mb steps throughout the autosomal genome were calculated. Thereinafter, we prospected genes mapped within genomic regions falling out the 5–95th the percentile using the *Biomart* tool of *Ensembl* (Flicek et al., 2013) (www.ensembl.org). Further, we performed an Overrepresentation Enrichment Analysis (ORA) to determine if the overrepresentation of gene ontology (GO) terms differed between the two tails of the empirical distribution of recombination rates. We used the WEB-based Gene SeT AnaLysis Toolkit (www.webgestalt.org) using the *Homo sapiens* and *Bos taurus* annotation databases and with the complete genome as the reference set.

## Results and Discussion

**Figure 1:**
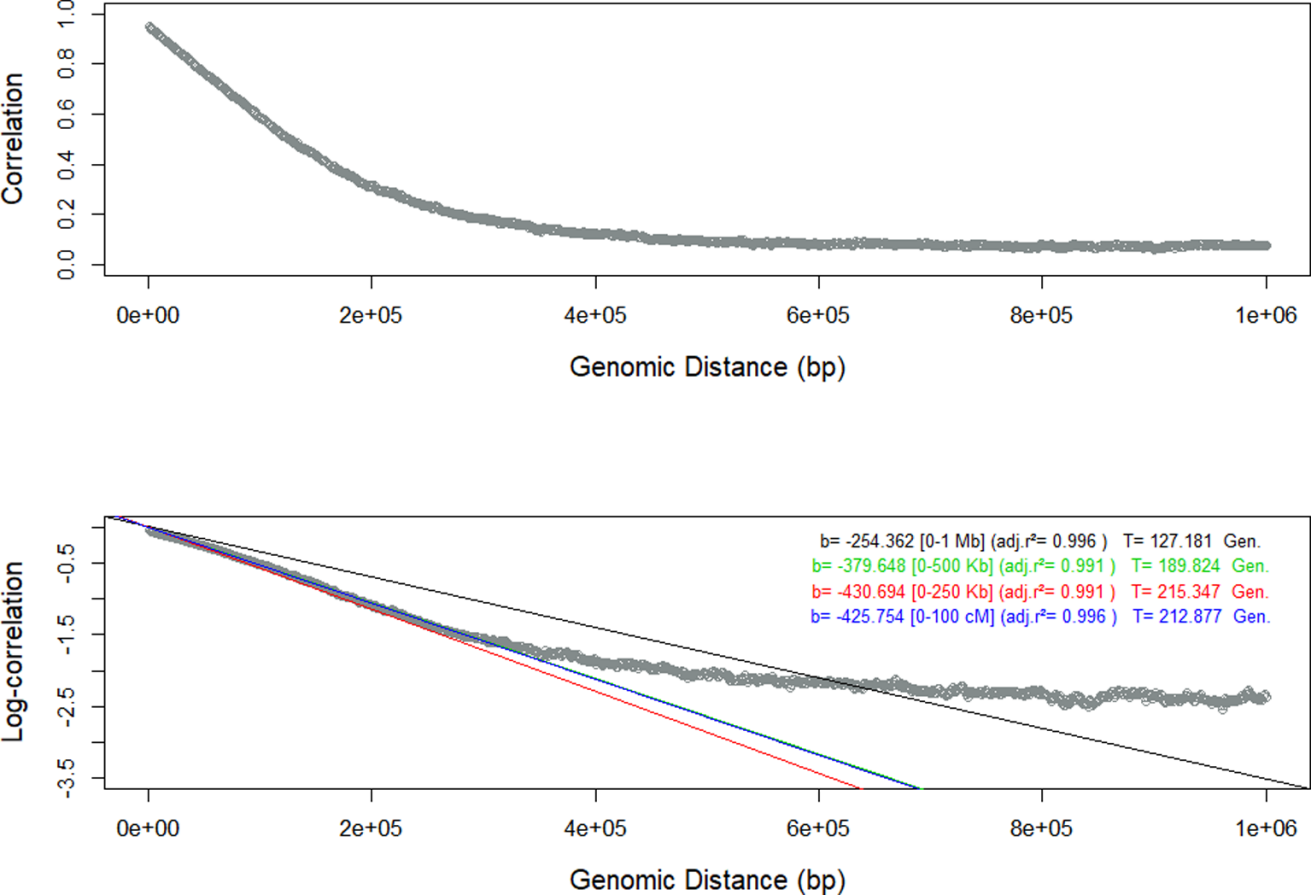
Persistency of Linkage Disequilibrium (LD) measured as the correlation and the log-correlation between the estimates of LD in Retinta (Re) and Rubia Gallega (RG) populations, and estimates of the slope of the regression with respect to the genomic distance for different ranges [0-1 Mb – black, 0-500 Kb-green, 0-250 Kb- red, and 0-100 Kb-blue].

We have used the persistency of LD phase among seven closely related populations (Cañas-Álvarez et al., 2015) to infer the landscape of the recombination events in a sequential procedure that involved several steps. As in other studies in livestock populations (Brito et al., 2015; Biegelmeyer et al., 2016; Brito et al., 2017; Grossi et al., 2017), the similitude of LD among populations was very high between adjacent markers and decreased rapidly with genomic distance. In the first step, we used all the SNP markers to estimate the persistency of LD phase for each pair of populations as the slope of the regression analysis between the natural logarithm of the correlations between the *r* measures of LD in bins of 20 kb on the genomic distance. Theoretically, this relationship should be linear (Hill and Robertson, 1968; De Roos et al., 2008). However, the results varied according to the genomic distance evaluated (1 Mb, 500 Kb, 250 Kb, or 100 Kb). To illustrate this phenomenon, the results of the regression analysis between Re and RG are shown in **Figure 1**. The regression analysis of the data within a range of 250 Kb had the highest adjusted R^2^ (0.999) and the linear relationship between persistency of LD phase and genomic distance was evident only in those first 250 Kb. The results for the other population pairs were similar (**Supplementary Information**, **Figures S1** to **S20**) and all of them had the highest adjusted R^2^ (> 0.998) with the first 250 Kb. In all population pairs, persistency of LD phase decayed rapidly over short distances, but in larger genomic distances remained > 0, as observed by De Roos et al. (2008). This is probably due to the fact that the populations were not totally divergent or due to the presence of some migration between them (De Roos et al., 2008) and consistent with a decrease in effective population size in cattle (Hayes et al., 2003).

In a second step, we restricted the analysis to the LD within 250 Kb and the slope of the regression analyses were equated to -2*c*T, with *c* set to 1.25 cM per Mb (Arias et al., 2009). We obtained 21 estimates of the number of generations of divergence (T) between populations (**Table 2**), that ranged from 132.3 (AV-BP) to 281.9 (Pi-Re). Estimates were in concordance with the results obtained by Cañas-Álvarez et al. (2016) for the same populations and dataset, and by De Roos et al. (2008) between two dairy populations (Holstein-Friesian and Jersey). However, the divergence times found in our study were lower than the observed between a dairy (Holstein) and beef (Angus) populations (De Roos et al., 2008).

**Table 2:** Estimated number of generations of divergence between seven beef cattle populations in Spain [Asturiana de los Valles (AV), Avileña - Negra Ibérica (ANI), Bruna dels Pirineus (BP), Morucha (Mo), Pirenaica (P), Retinta (Re), Rubia Gallega (RG)] based on the architecture of Linkage Disequilibrium.

	ANI	BP	Mo	Pi	Re	RG
AV	181.2	132.3	160.7	184.1	185.9	157.2
ANI	–	244.9	133.6	268.6	175.1	225.1
BP	–	–	232.8	168.1	258.1	176.4
Mo	–	–	–	252.8	168.8	205.2
Pi	–	–	–	–	281.9	215.3
Re	–	–	–	–	–	229.1

The estimates of T assumed that the variance of the LD (r) remained constant in each population and this is probably far from the truth. The effective size of the populations has decreased in the last generations (Cañas-Alvarez et al., 2016) and, thus, the estimates of T are probably overestimated. Nevertheless, the bias caused by the variations in the effective size should be similar throughout the autosomal genome and, therefore, local variations in the persistency of LD phase are informative for the inference of the recombination landscape along the autosomal genome.

After this preliminary step, we used the same expression (-2*c*T) to infer the distribution of the recombination rate *c*. Now, the numbers of generations of divergence (T) were assumed to be known and they were replaced by their estimates with all the SNP markers. In this case, we equated the slope of the regression of the persistency of LD phase on the genomic distance to -2*c*T, but the analysis was restricted to the SNP markers located within a sliding window of 1 Mb (500 kb downstream and 500 kb upstream) in steps of 0.1 Mb. We obtained as many as 25,098 estimates of *c* for each pair of populations (**Figures S21** to S41). The rationale to this approach was that genetic drift and variations on the effective size of the populations should have affected the entire autosomal genome with the same intensity and, therefore, regional variation in the persistency of LD phase should reflect variations in the recombination rate. However, as in other LD-based procedures for estimating the recombination rate (Li and Stephens, 2003), it could also reflect differences in the intensity of the mutation rate or the occurrence of selection events.

**Figure 2:**
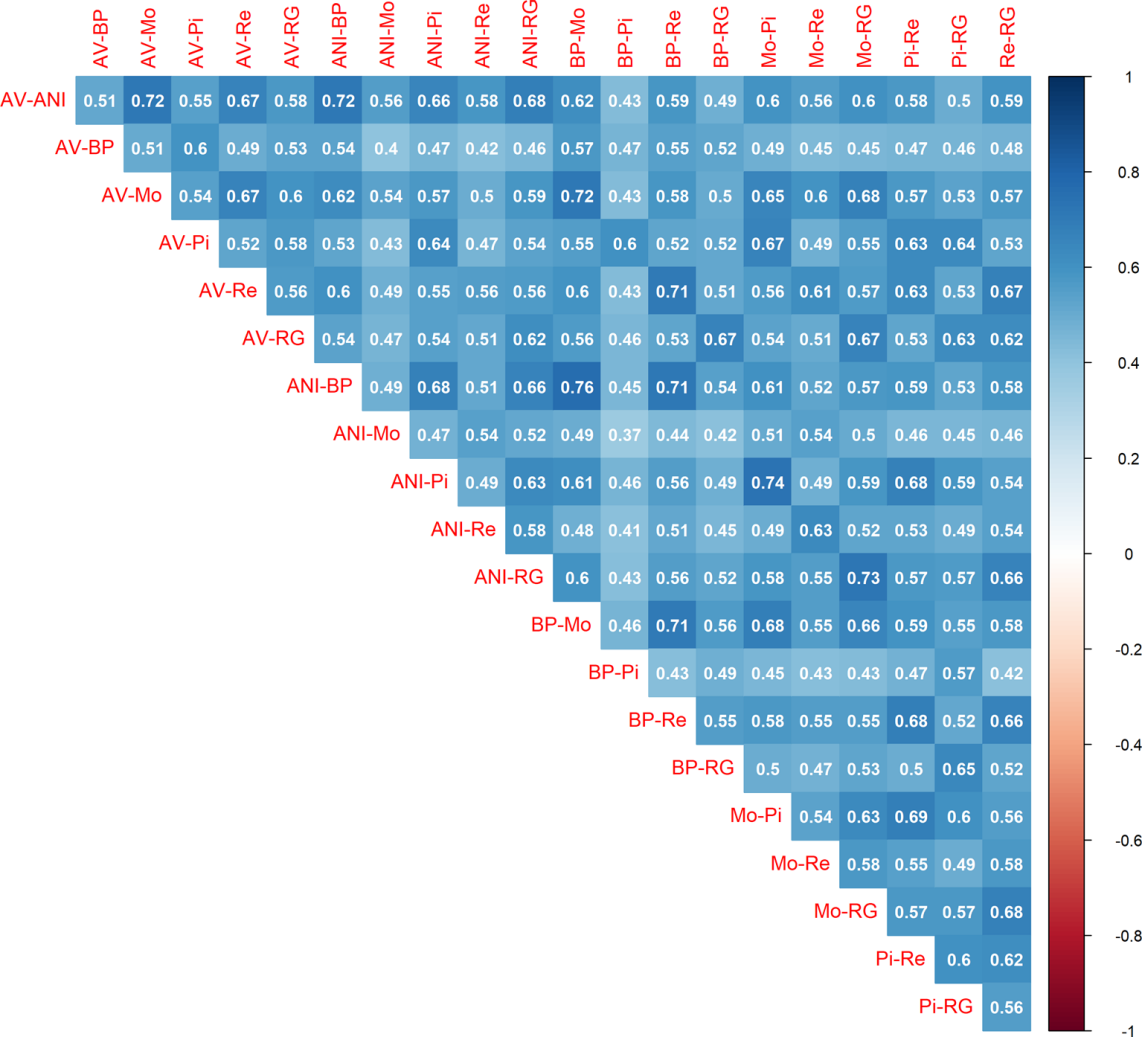
Correlations between the estimates of the recombination rate (c) in bins of 1 Mb between pairs of seven beef cattle populations in Spain [Asturiana de los Valles (AV), Avileña - Negra Ibérica (ANI), Bruna dels Pirineus (BP), Morucha (Mo), Pirenaica (P), Retinta (Re), Rubia Gallega (RG)].

Once the local estimates of *c* for the 21 pair of populations were available, we calculated the correlations between them (**Figure 2**). Values ranged from moderate (0.37) to strong (0.76), with an average of 0.55 and a standard deviation of 0.08. The results were consistent with the output of **Table 2**. The minimum correlation (0.37) was obtained between ANI-Mo and BP-Pi, which is consistent with the large number of generations of divergence between ANI and BP (244.9), ANI and Pi (268.6), Mo and BP (232.8), and Mo and Pi (252.8). In contrast, the greatest correlation was between ANI-BP and Mo-BP, given that the number of generations of divergence between ANI and Mo is only 133.6 generations. Therefore, given the noise in the LD estimates with small sample sizes, this average correlation and the consistency of estimates between pairs of populations should be considered very relevant. The similarity between estimates was within the same range than the reported between estimates of the recombination rate in human (Graffelman et al., 2007; Laayouni et al., 2011; Manu et al., 2018) and livestock (Petit et al., 2017; Shen et al., 2018) populations. Thus, somehow it confirms that similarities in the distribution of the recombination rate are achieved between closely related populations (Smukowski and Noor, 2011), such as the analyzed in this study (Cañas-Álvarez et al., 2015; González-Rodríguez et al., 2017). This similarity was even observed between pairs of populations that do not share any population (i. e. ANI-AV and BP-Pi), whose average correlation (0.52 ± 0.06) was only slightly lower than the average of the correlations between estimates from pairs that share (i.e. ANI-AV vs ANI-Mo) (0.58 ± 0.08). It reinforces the hypothesis that the similarity between persistence of LD phase at different locations of the genome and between pairs of populations is related with variations in the recombination rate throughout the genome.

**Figure 3:**
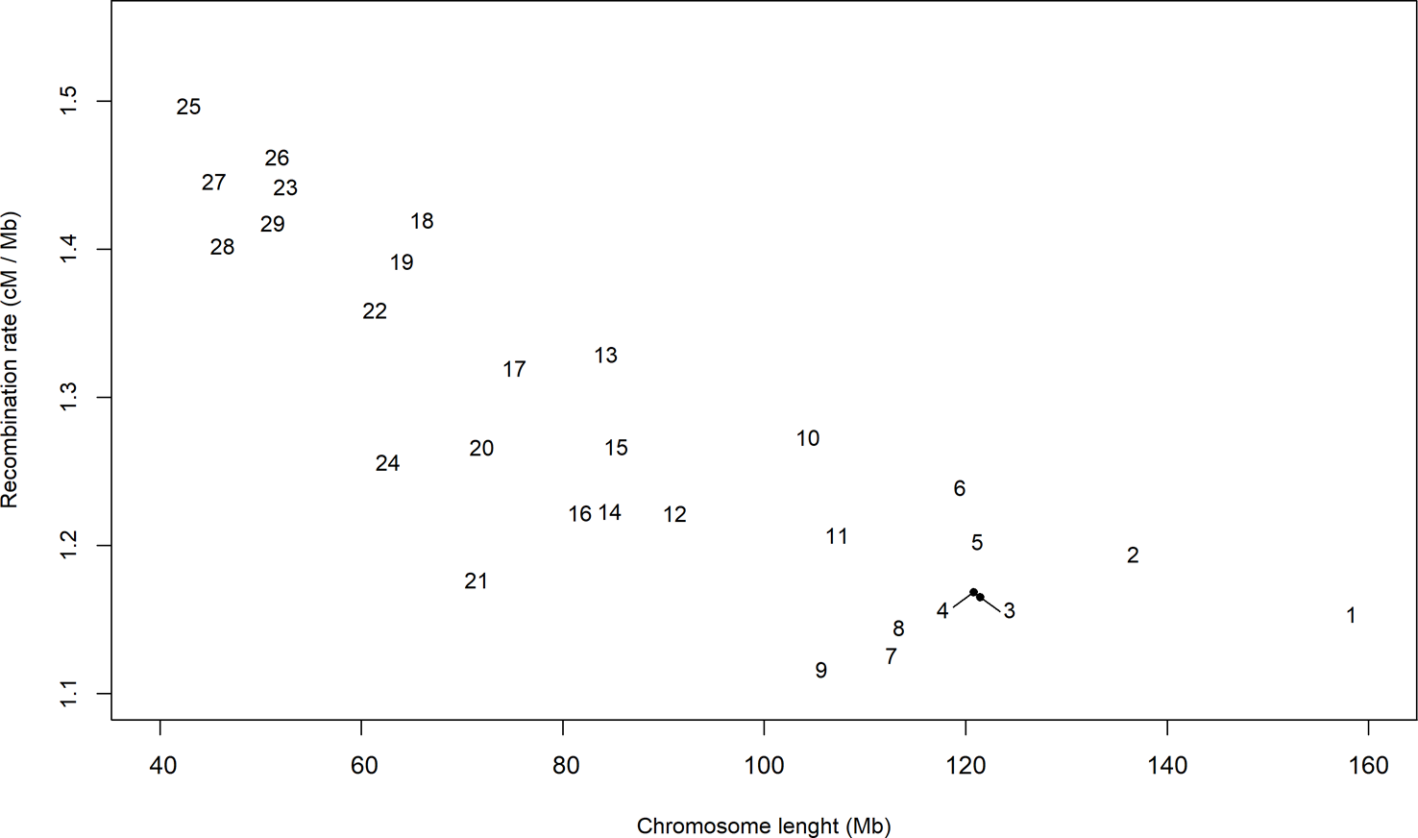
Average estimate per chromosome of the recombination rate (cM/Mb) in bins of 1 Mb and for all pairs of seven breeds of beef cattle populations in Spain.

Despite divergences associated with a specific population pair and possible selection events, the distribution of the persistency of LD phase appeared to follow a global pattern. Therefore, we used the estimates of *c* to describe the distribution of the recombination rate along the autosomal genome. Initially, we calculated an average of all of the estimates of *c* for each chromosome (**Figure 3**), which ranged from 1.12 cM per Mb in BTA9 to 1.50 cM per Mb in BTA25. In general, the largest chromosomes tended to have the lowest recombination rates. The relationship between recombination rate and chromosome length (Kaback et al., 1992; Jensen-Seaman et al., 2004; Li and Freudenberg, 2009) or genome length (Lynch, 2006) has been reported in several species and may be associated to the difficulties of small chromosomes to find their homologues during meiosis (Tiemann-Boege et al., 2017). In fact, Fledel-Alon et al. (2009) suggested that, in meiosis, each chromosome usually undergoes at least one crossing-over, which produces a very strong correlation between the average number of crossovers and chromosome length.

**Figure 4:**
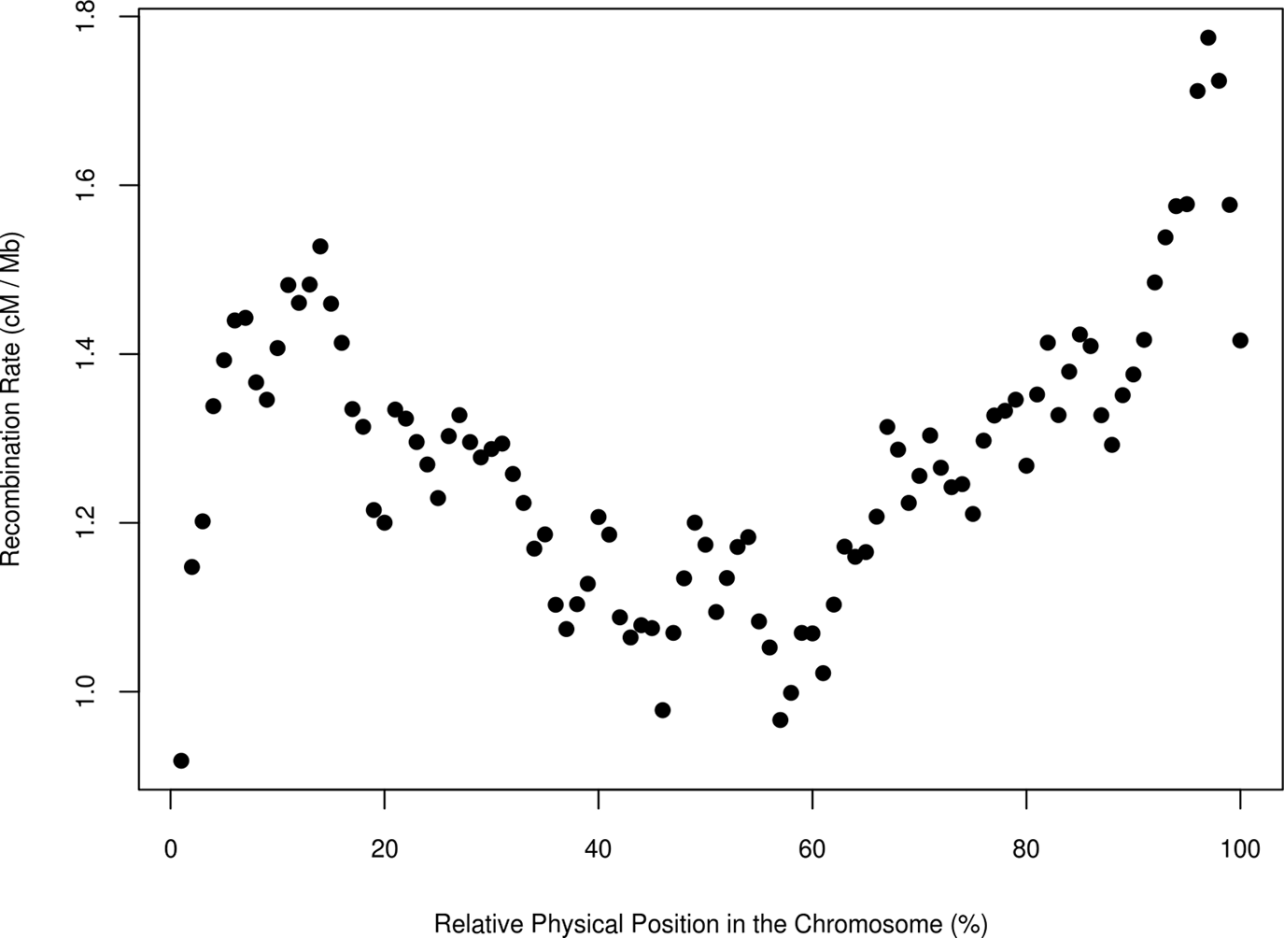
Average estimate of the recombination rate (cM/Mb) in bins of 1 Mb and for all pairs of populations and the relative physical position in the chromosome in seven breeds of beef cattle in Spain.

Next, we evaluated the relationship between the recombination rate and the relative physical position within the chromosome by averaging the *c* estimates for each percentile along the length of each chromosome (**Figure 4**). The results were similar to those of Sandor et al. (2012); Ma et al. (2015), and Shen et al. (2018) in the bovine specie and to the results of haplotype diversity measured in the same populations (Mouresan et al., 2017). In cattle, all autosomal chromosomes are acrocentric (Popescu, 1990) and, in our study, the recombination rate was lowest at the beginning of the chromosome, near the centromere. Furthermore, a low recombination rate was evident at the middle of the chromosome, although the centromere of chromosomes in cattle is not located there. Ma et al. (2015) argued that the bimodal distribution of recombination rates might be caused by positive crossover interference. The highest recombination rate was at the distal portion of the chromosome (over the 95 % percentile of the relative position within chromosomes), in agreement with studies that have shown that recombination rate is highest at the telomeres (Nachman, 2002; Coop and Przeworski, 2007).

**Figure 5:**
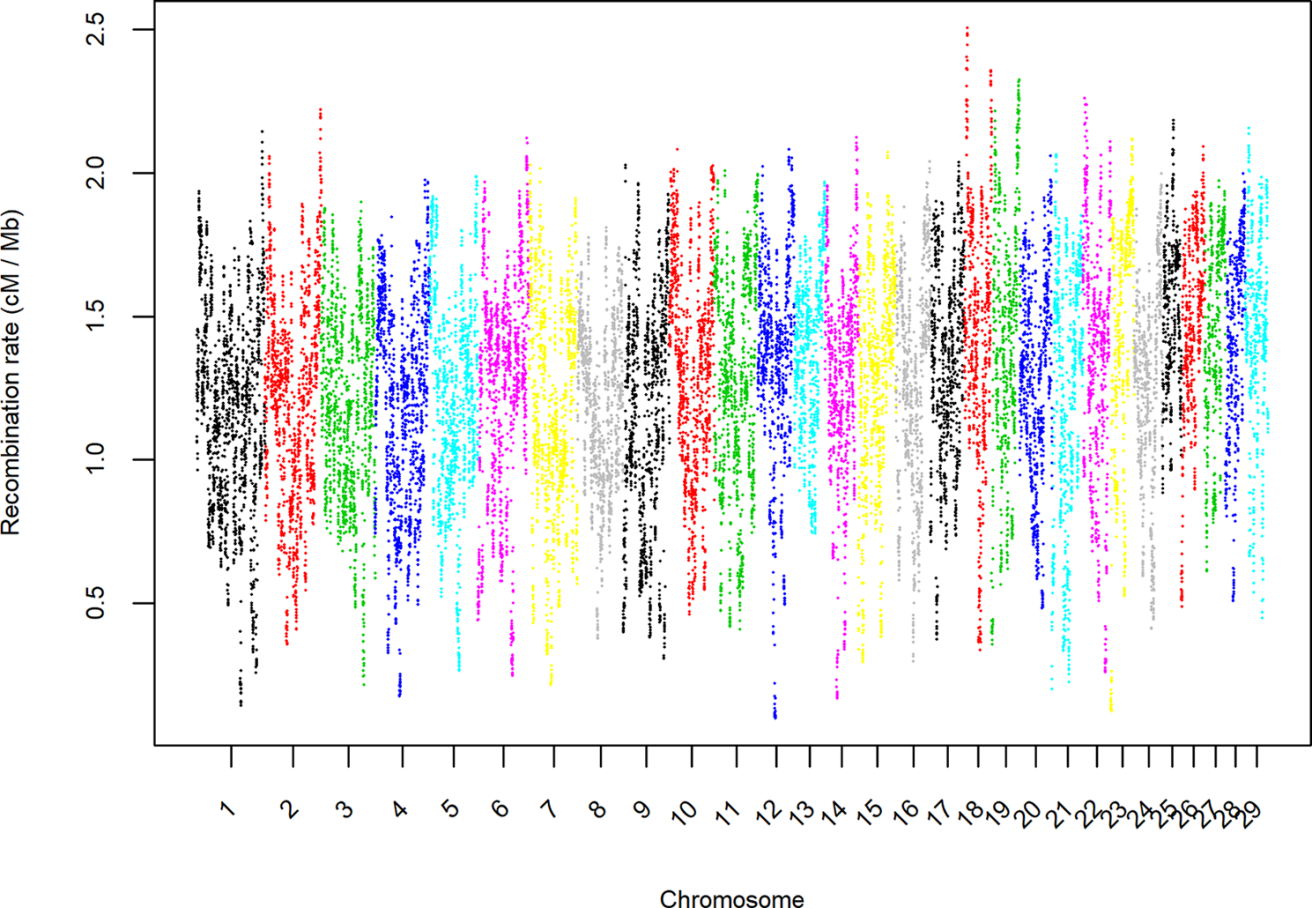
Average estimate of the recombination rate (cM/Mb) in bins of 1 Mb and throughout the autosomal genome for all pairs of seven beef cattle populations in Spain.

**Figure 6:**
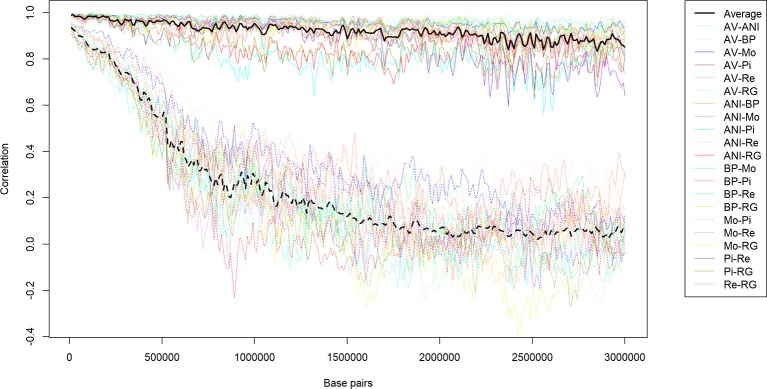
Average (bold lines) and specific population pair (transparent lines) persistency of LD (correlation between LD estimates in pairs of populations) in the genomic regions that had the highest (BTA12-42.4 Mb, continuous lines) or the lowest persistency (BTA18-7,1 Mb, dotted lines) among seven beef breeds in Spain.

Additionally, a map of the recombination rates throughout the genome was calculated by averaging the estimates from the 21 pairs of populations in 0.1 Mb steps along the autosomal chromosomes (**Figure 5**). The average estimate of the recombination rate was 1.275 cM per Mb with a standard deviation of 0.381. As expected, the recombination rate was very similar to the rate assumed in the initial step of the study (1.25 cM per Mb). The estimated recombination rates within the genome were highly heterogeneous, and the ratio between the genomic regions that had the highest (BTA18-7.1 Mb) and the lowest (BTA12-42.4 Mb) estimated recombination rates was 25.37. To illustrate the differences between those two extreme regions of the genome, **Figure 6** displays the average recombination rate and all specific persistencies of LD phase for each population pair.

The heterogeneity of the recombination rate reflected the presence of highly recombining genomic regions. Most of the recombination events may occur in a small portion of the genome, as observed in other species. However, the Gini index between the cumulative distributions of the recombination rate and the genetic distance was 0.1803, which is lower than others reported in human (Kong et al., 2002), apes (Stevison et al., 2016), and livestock (Petit et al., 2017) populations. It is likely that the Gini index was low because the method used in our study was only able to distinguish among the rates of recombination (as a measure of the persistency in LD phase) within relatively large genomic regions (1 Mb), and recombination hotspots often are restricted to 1–2 kb (Myers et al., 2005; Mancera et al., 2008).

The 5^th^ and 95^th^ percentiles of the recombination rate estimates were 0.593 and 1.856 cM per Mb, respectively, and we identified the genes within the genomic regions that had values that were either above or below those percentiles. The number of genes within the regions that were above the 95^th^ (high recombination rate – HRR-) or below the 5th (low recombination rate –LRR-) percentiles were 665 and 669, respectively. Some studies have suggested that there is a negative correlation between gene density and the frequency of recombination hotspots (Myers et al., 2005; Freudenberg et al., 2009; Stapley et al., 2017), which was not detectable with the methods used in our study.

Furthermore, some authors (The International HapMap Consortium, 2007) have suggested that genes with highly conserved function are located surrounding regions with low recombination rate; on the other hand, HRR regions contains genes that are exposed to recurrent adaptive process to allow plasticity of organism to coming circumstances. In our study, we have tried to corroborate these statements using ORA with the GO terms for biological processes, cellular components, and molecular functions. The results are presented in **Tables 3** to **5** (*Homo sapiens* annotation database) and in **Supplementary Tables 1 to 3** (*Bos taurus* annotation database). In general, the results with the *Homo sapiens* database yielded results with lower FDR than with the *Bos taurus* database, probably because the human genome in notably more annotated than the bovine one.

**Table 3:** False discovery rate (FDR) for the top 10 enriched Gene Ontology (GO) terms for Biological processes with the Homo sapiens database for genes within the genomic regions located over the 95th (high recombination rate) and below the 5th (low recombination rate) percentiles of the average recombination rates.

High Recombination Rate		Low Recombination Rate	
**GO TERM**	**FDR**	**GO TERM**	**FDR**
Protein citrullination	2.00e-04	Homophilic cell adhesion via plasma membrane adhesion molecules	2.45e-01
Histone citrullination	2.00e-04	DNA replication initiation	2.45e-01
Extracellular vesicle biogenesis	3.09e-01	Vitamin A metabolic process	2.45e-01
Regulation of action potential	3.27-01	Cell-cell adhesion via plasma-membrane adhesion molecules	5.84e-01
Regulation of substrate adhesion-dependent cell spreading	3.27-01	Regulation of chemokine biosynthetic process	5.84e-01
Sodium ion transmembrane transport	3.27-01	Celullar response to electrical stimulus	5.84e-01
Peptidyl-arginine modification	3.27-01	Chemokine biosynthetic process	5.84e-01
phagosome acidification	3.27-01	Chemokine metabolic process	5.84e-01
Cardiac muscle cell action potential	3.27-01	Neutrophil mediated killing of bacterium	1.00e-00
Regulation of fibroblast growth factor receptor signaling pathway	3.27-01	Sulfur amino acid catabolic process	1.00e-00

**Table 4:** False discovery rate (FDR) for the top 10 enriched Gene Ontology (GO) terms for Cellular Components with the Homo sapiens database for genes within the genomic regions located over the 95th (high recombination rate) and below the 5th (low recombination rate) percentiles of average recombination rates.

High Recombination rate		Low Recombination Rate
GO TERM	FDR	GO TERM
Neuron part	0.02e-04	Plasma membrane region
Synapse	0.02e-04	Golgi apparatus
Synapse part	1.5e-03	Intrinsic component of the plasma membrane
Golgi apparatus	1.5e-03	Cytoplasmic vesicle part
Cell projection part	1.5e-03	Integral component of the plasma membrane
Plasma membrane bounded cell projection part	1.5e-03	Perinuclear region of cytoplasm
Postsynapse	3.0e-03	Cell projection part
Neuron projection	3.2e-03	Plasma membrane bounded cell projection part
Vacuole	3.7e-03	Golgi subcompartment
Phagocytic vesicle	4.8e-03	Plasma membrane protein complex

**Table 5:** False discovery rate (FDR) for the top 10 enriched Gene Ontology (GO) terms for Molecular Functions for genes with the Homo sapiens database within the genomic regions located over the 95th (high recombination rate) and below the 5th (low recombination rate) percentiles of the average recombination rates.

High Recombination Rate		High Recombination Rate	
GO TERM	FDR	GO TERM	FDR
Protein-arginine deaminase activity	1.00e-04	GTP-dependent protein binding	8.01-0e2
Hydrolase activity, acting on carbon nitrogen (but not peptide) bonds, in linear amidines	1.13e-02	Aminoacyl-tRNA ligase activity	4.76e-01
Solute: cation antiporter activity	4.74e-02	Ligase activity, forming carbon-oxigen bonds	4.76e-01
Cation: cation antiporter activity	7.49e-02	Molecular carrier activity	4.76e-01
Potassium: proton antiporter activity	1.01e-01	Drug binding	4.76e-01
Metal ion transmembrane transported activity	1.11e-01	Phosphatidylinositol bisphosphate kinase activity	4.76e-01
Solute: Proton antiporter activity	1.16e-01	ARF guanyl-nucleotide exchange factor activity	4.76e-01
Monovalent cation: proton antiporter activity	1.16e-01	Nucleocytoplasmic carrier activity	4.76e-01
Sodium: proton antiporter activity	1.16e-01	Identical protein binding	4.76e-01
Monovalent inorganic cation transmembrane transported activity	1.19e-01	Metalloexopeptidase activity	4.76e-01

The results of the enrichment analysis for biological processes with the human database (**Table 3**) only provided enriched GO terms with a False Discovery Rate (FDR) lower than 0.05 with the genes present in the HRR genomic regions. The significant GO terms correspond to *Protein citrullination* and *Histone citrullination*. Citrullination, the conversion of the amino acid arginine in a protein into the amino acid citrulline, has been related to an increase in antigenic diversity (Nguyen and James, 2016). The higher recombination rate of those genomic regions might indicate that they have evolved to have high plasticity to adapt to changing environments (Charlesworth et al., 2009; Campos et al., 2014). Therefore, the generation of new genetic variants by recombination may help the antigen diversity from the perspective of the host. Thus, it works as a mechanism to adapt its immune response to fight against the ability of the pathogen to modify its antigenic targets. The results obtained from the *Bos taurus* database (**Supplementary Table 1**) were not significant (FDR < 0.05).

The top 10 enriched GO terms for cellular components for the genomic regions that had either high or low recombination rate are presented in **Table 4** (*Homo sapiens*) and **Supplementary Table 2** (*Bos taurus*). For *Homo sapiens*, the FDR was generally lower than it was for biological processes (FDR < 4.6e-02). Some cellular components (*Golgi apparatus*, *Cell projection part, Plasma membrane bounded bell projection part*) occurred in both types of genomic regions, but there were some important differences between them. The HRR genomic regions were enriched with genes whose expression is located at the extracellular space and related with neuronal interactions (*neuron part, synapse, synapse part, postsynapse, or neuron projection*). In contrast, the genes located at LRR regions were associated with very basic intracellular (*Cytoplasmic vesicle part*, *Perinuclear region of the cytoplasm*) or membrane components (*Plasma membrane region*, *Intrinsic component of the plasma membrane Integral component of the plasma membrane*, *Plasma membrane protein complex*). The results obtained from the *Bos taurus* database were significant (FDR < 0.05) only for *cytosol* and *nuclear lumen* in the LRR regions, confirming the results provided by the *Homo sapiens* database.

The enriched GO terms for molecular functions are presented in **Table 5** (*Homo sapiens*) and **Supplementary Table 3** *(Bos Taurus*). The three significantly (FDR < 0.05) enriched GO terms for the HRR genomic regions were coherent with the enriched GO terms for biological processes. In fact, two of those were clearly associated with citrullination [*Protein-arginine deiminase activity* and *Hydrolase activity, acting on carbon nitrogen (but not peptide) bonds, in linear amidines*] and the other (*Solute: cation antiporter activity)* was linked to transmembrane transportation of solutes. In contrast, the only significantly enriched GO terms for LRR genomic regions was *GTP-dependent protein binding* (FDR = 8.01e-02), which was confirmed with the results from the *Bos taurus* database (FDR = 2.7e-02). The genes located in the LRR genomic regions should be more conserved, since they may be necessary for basic functions of the organism. In this sense, the genes belonging to the *GTP-dependent protein GO term* regulate guanine nucleotide-binding proteins that play a crucial role in signal transduction and in a large number of cellular processes (Zachariou et al., 2012).

The results of this study confirm that the genomic architecture of persistency of LD phase is well conserved among closely related populations, such as the Spanish autochthonous beef cattle breeds, and is heterogeneous within the autosomal genome and that this heterogeneity can be used to estimate the recombination rate. Several studies have estimated the persistency of LD phase between populations as a measure of genetic diversity (De Roos et al., 2008; Villa-Angulo et al., 2009; Cañas-Álvarez et al., 2016) and as a mean of predicting the marker density required for multi-breed genomic evaluation (De Roos et al., 2008; Cañas-Álvarez et al., 2016). Nevertheless, the genetic architecture of persistency of phase within the genome has received limited attention. In this study, we estimated the persistency of LD phase among seven beef cattle populations in Spain and its distribution within the genome, which has been related with the genetic architecture of the recombination rates. Even though the recombination rate varies among species, sex, and populations (Dapper and Payseur, 2017), some general patterns were described (Tiemann-Boege et al., 2017). The patterns were confirmed in our analysis by the similitude of our results with some studies in other species or populations (Ma et al., 2015; Shen et al., 2018).

Therefore, the main conclusion of this study is that the heterogeneity of persistency of LD phase between closely related populations can be used to estimate the recombination rate with the procedure developed here, which is simpler and brings similar results as more complex and coalescent dependent methods. This implies that it may help to identify regions related to hot and cold spots when data from several populations of the same ancestral origin are available. Nevertheless, our procedure has several limitations since differences in the mutation rate or selection events can locally affect the persistency of LD phase and it requires populations to be close enough that the recombination rate is well conserved.

## Author Contributions

The study was conceived by LV. Preliminary data preparation was done by EM, AG-R, JC-Á, and SL. Data analysis was done by EM and LV. EM and LV produced the draft manuscript. JA, CD, AM, and JP collaborated in generating the data. SL, JA, CD, JB, AM, PL-B, and JP discussed the results. All authors reviewed and revised the manuscript.

## Funding

We thank the AGL 2010-15903 grant from the Spanish government and the KBBE.2011.1.3-06 project from the European Union’s Seventh Framework. We also thank the Breed Societies for their collaboration in collecting samples. The support of FEAGAS is acknowledged. JC-Á acknowledges the COLCIENCIAS support by the Francisco José De Caldas Fellowship 497/2009, and AG-R acknowledges the financial support provided by the BES-2011-045434 fellowship.

## Conflict of Interest

The authors declare that the research was conducted in the absence of any commercial or financial relationships that could be construed as a potential conflict of interest.
