# Supplementary material for: Mapping Recombination Rate on the Autosomal Chromosomes Based on the Persistency of Linkage Disequilibrium Phase Among Autochthonous Beef Cattle Populations in Spain
Source: Front Genet. 2019 Nov 20;10:1170. doi: 10.3389/fgene.2019.01170 (PMC6880760; doi:10.3389/fgene.2019.01170)

Supplementary Table 1. *False discovery rate (FDR) for* the *top 10 enriched GO terms for Biological processes with the Bos taurus database for genes within the genomic regions located over the 95th (high recombination rate) and below the 5th (low recombination rate) percentiles of average recombination rates*.

High Recombination Rate		Low Recombination Rate	
GO TERM	FDR	GO TERM	FDR
inorganic cation import across plasma membrane	5.97e-01	RNA splicing, via transesterification reactions	6.61e-01
inorganic ion import across plasma membrane	5.97e-01	RNA splicing, via transesterification reactions with bulged adenosine as nucleophile	6.61e-01
regulation of cellular macromolecule biosynthetic process	7.15e-01	mRNA splicing, via spliceosome	6.61e-01
regulation of RNA metabolic process	7.15e-01	DNA replication initiation	1.00e-00
regulation of gene expression	7.15e-01	mRNA processing	1.00e-00
import across plasma membrane	7.15e-01	cellular response to ketone	1.00e-00
regulation of cellular biosynthetic	7.15e-01	cell part morphogenesis	1.00e-00
regulation of biosynthetic process	7.15e-01	locomotory behavior	1.00e-00
regulation of macromolecule biosynthetic process	7.15e-01	plasma membrane bounded cell projection morphogenesis	1.00e-00
Regulation of nucleobase-containing compound metabolic process	7.15e-01	cell morphogenesis involved in neuron differentiation	1.00e-00

Supplementary Table 2. *False discovery rate (FDR) for the top 10 enriched GO terms for Cellular Components with the Bos taurus database for genes within the genomic regions located over the 95th (high recombination rate) and below the 5th (low recombination rate) percentiles of the average recombination rates.*

High Recombination rate		Low Recombination rate	
GO TERM	FDR	GO TERM	FDR
phagocytic vesicle	5.24e-02	cytosol	6.79e-04
cytosol	3.37e-01	nuclear lumen	1.03e-04
glutamatergic synapse	4.51e-01	nucleoplasm	1.04e-01
endocytic vesicle	4.51e-01	endoplasmic reticulum	1.04e-01
transcription factor complex	4.51e-01	intrinsic component of external side of plasma membrane	1.68e-01
anchored component of membrane	4.51e-01	anchored component of external size of plasma membrane	1.68e-01
tethering complex	4.51e-01	golgi apparatus	1.94e-01
cytoplasmic dynein complex	6.11e-01	ubiquitin ligase complex	3.56e-01
nucleoplasm	6.11e-01	cullin-RING ubiquitin ligase complex	3.56e-01
nuclear lumen	6.11e-01	Sm-like protein family complex	4.11e-01

Supplementary Table 3. *False discovery rate (FDR) for the top 10 enriched GO terms for Molecular Functions for genes with the Bos Taurus database within the genomic regions located over the 95th (high recombination rate) and below the 5th (low recombination rate) percentiles of the average recombination rates*.

High Recombination Rate		Low Recombination Rate	
GO TERM	FDR	GO TERM	FDR
hydrolase activity, acting on carbon-nitrogen (but not peptide) bonds, in linear amidines	3.22e-01	GTP-dependent protein binding	2.7e-02
cation: cation antiporter activity	1.00e-00	mRNA binding	3.78e-01
solute: cation antiporter activity	1.00e-00	5S rRNA binding	9.49e-01
calmodulin binding	1.00e-00	aminoacyl-tRNA ligase activity	1.00e-00
RNA polymerase II activating transcription factor binding	1.00e-00	ligase activity, forming carbon-oxygen bonds	1.00e-00
phosphatidylinositol 3-kinase regulatory subunit binding	1.00e-00	DNA replication origin binding	1.00e-00
lysine-acetylated histone binding	1.00e-00	carbonate dehydratase activity	1.00e-00
acetylation-dependent protein binding	1.00e-00	RNA binding	1.00e-00
voltage-gated potassium channel activity	1.00e-00	metallochaperone activity	1.00e-00
mRNA binding	1.00e-00	sialic acid transmembrane transporter activity	1.00e-00

Supplementary Figure 1. *Persistency of Linkage Disequilibrium (LD) measured as the log-correlation between estimates of LD between populations of Asturiana de los Valles – AV- and Avileña Negra-Ibérica -ANI- and estimates of the slope of the regression with respect to the genomic distance for different ranges [0-1 Mb – black, 0-500 Kb-green, 0-250 Kb- red and 0-100 Kb-blue].*

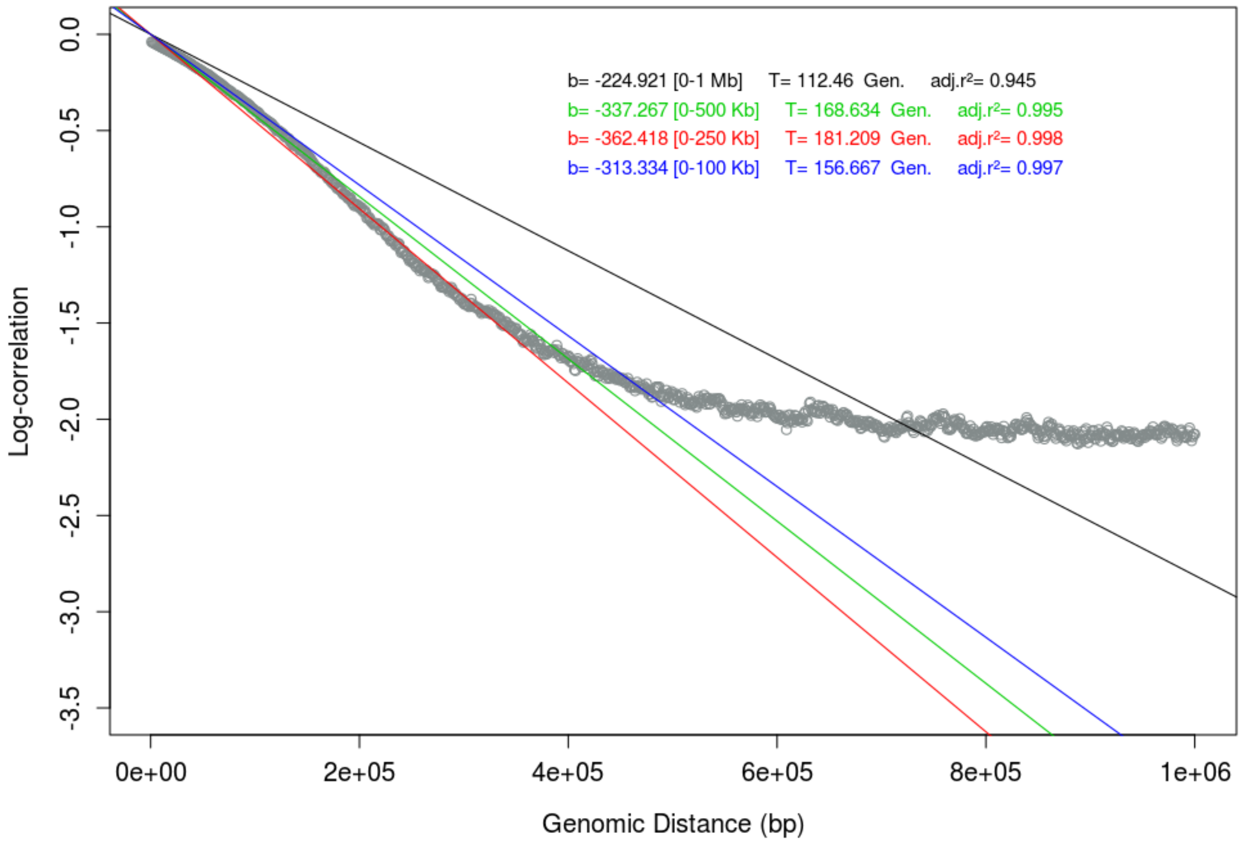

Supplementary Figure 2. *Persistency of Linkage Disequilibrium (LD) measured as the log-correlation between estimates of LD between populations of Asturiana de los Valles – AV- and Bruna dels Pirineus -BP- and estimates of the slope of the regression with respect to the genomic distance for different ranges [0-1 Mb – black, 0-500 Kb-green, 0-250 Kb- red and 0-100 Kb-blue].*

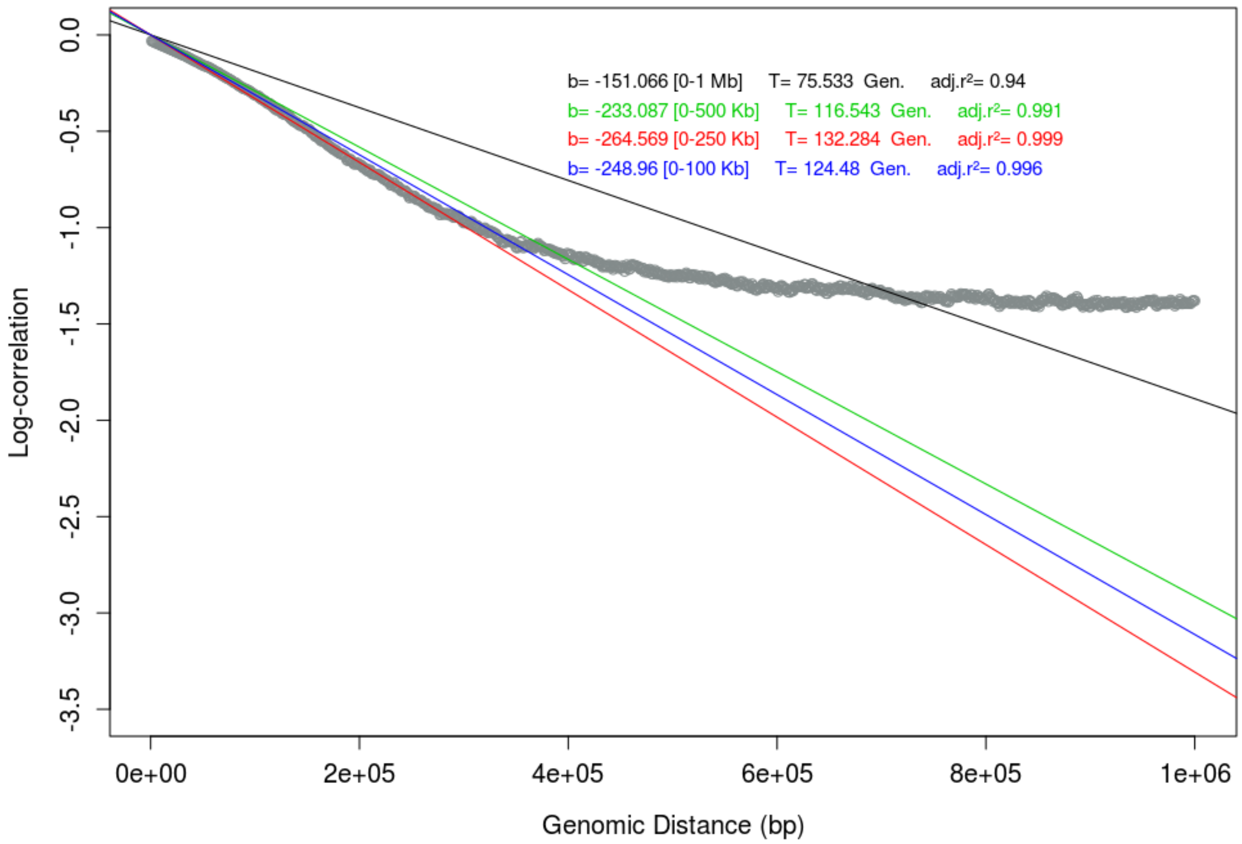

Supplementary Figure 3. *Persistency of Linkage Disequilibrium (LD) measured as the log-correlation between estimates of LD between populations of Asturiana de los Valles – AV- and Morucha -Mo- and estimates of the slope of the regression with respect to the genomic distance for different ranges [0-1 Mb – black, 0-500 Kb-green, 0-250 Kb- red and 0-100 Kb-blue].*

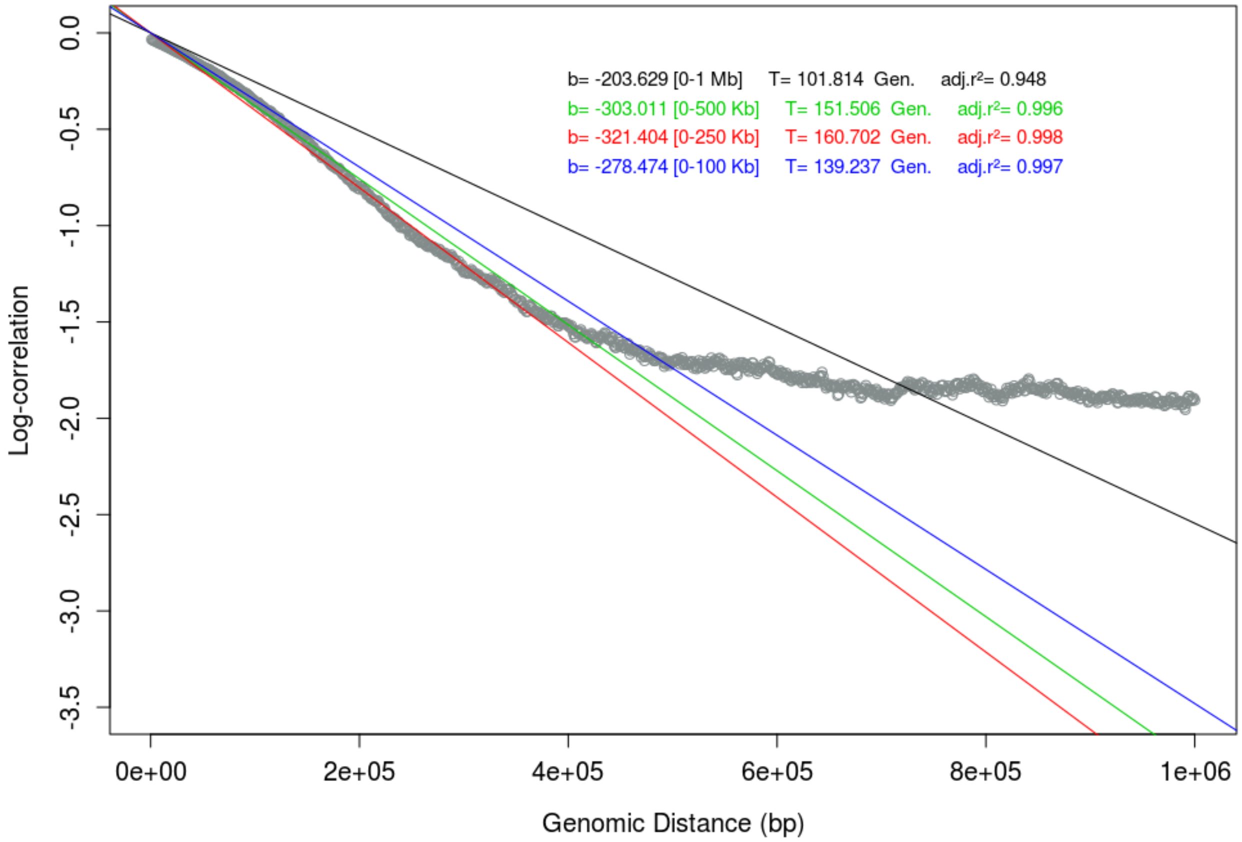

Supplementary Figure 4. *Persistency of Linkage Disequilibrium (LD) measured as the log-correlation between estimates of LD between populations of Asturiana de los Valles – AV- and Pirenaica -Pi- and estimates of the slope of the regression with respect to the genomic distance for different ranges [0-1 Mb – black, 0-500 Kb-green, 0-250 Kb- red and 0-100 Kb-blue].*

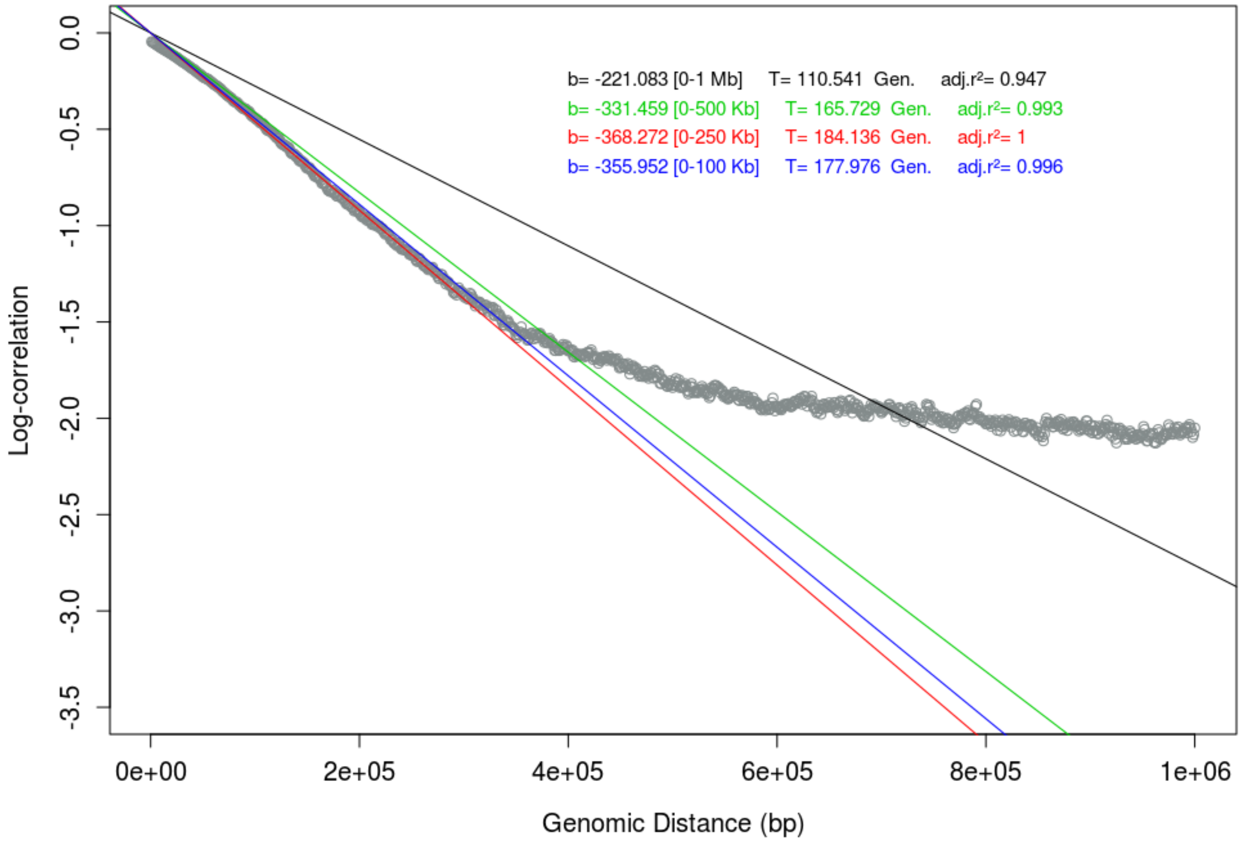

Supplementary Figure 5. *Persistency of Linkage Disequilibrium (LD) measured as the log-correlation between estimates of LD between populations of Asturiana de los Valles – AV- and Retinta -Re- and estimates of the slope of the regression with respect to the genomic distance for different ranges [0-1 Mb – black, 0-500 Kb-green, 0-250 Kb- red and 0-100 Kb-blue].*

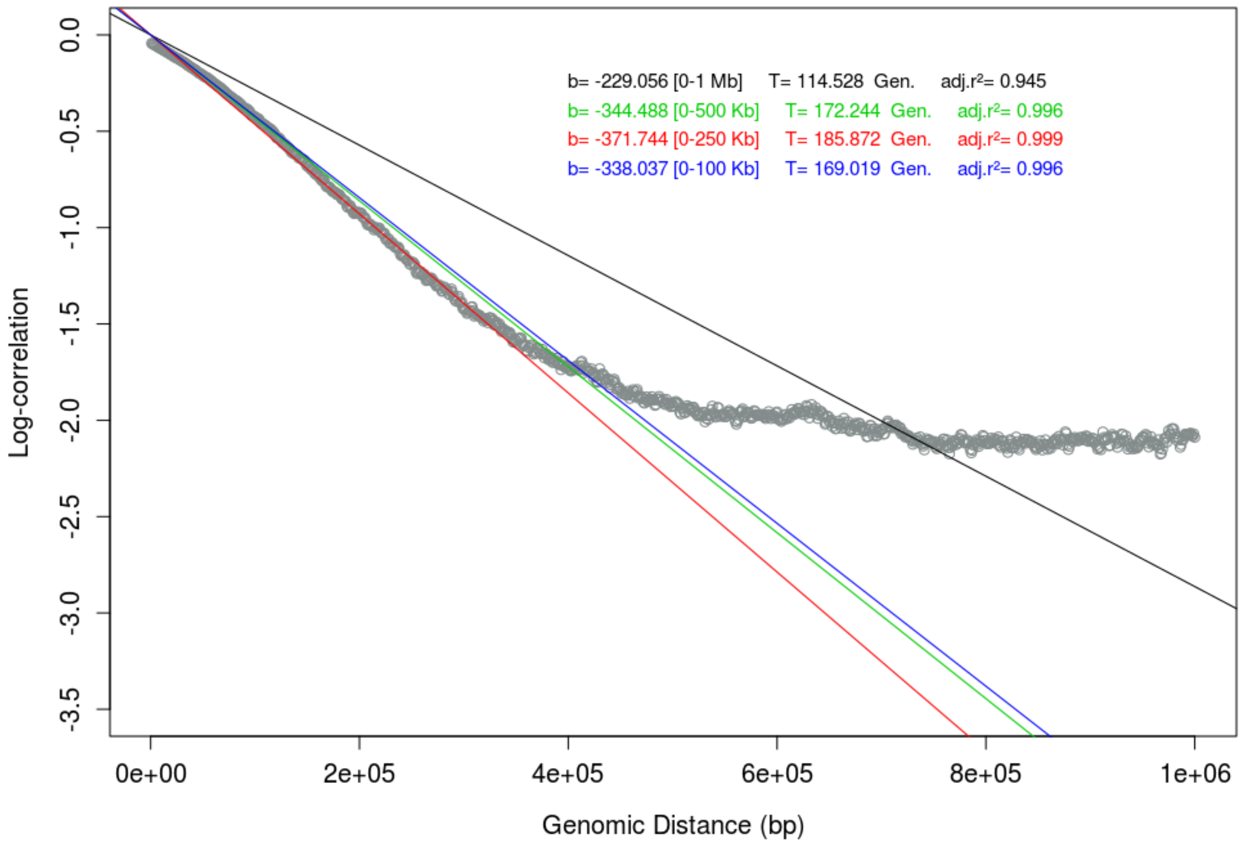

Supplementary Figure 6. *Persistency of Linkage Disequilibrium (LD) measured as the log-correlation between estimates of LD between populations of Asturiana de los Valles – AV- and Rubia Gallega -RG- and estimates of the slope of the regression with respect to the genomic distance for different ranges [0-1 Mb – black, 0-500 Kb-green, 0-250 Kb- red and 0-100 Kb-blue].*

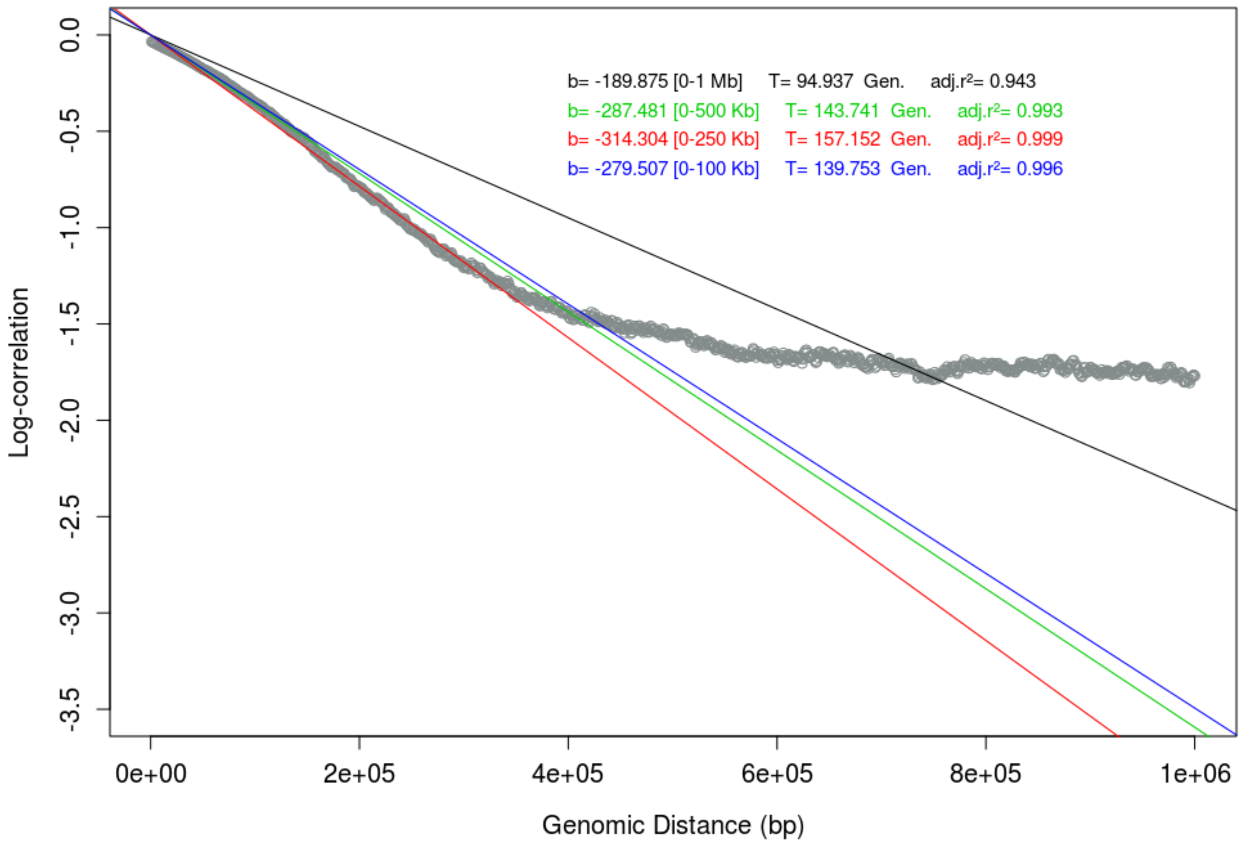

Supplementary Figure 7. *Persistency of Linkage Disequilibrium (LD) measured as the log-correlation between estimates of LD between populations of Avileña Negra-Ibérica -ANI- and Bruna dels Pirineus -BP- and estimates of the slope of the regression with respect to the genomic distance for different ranges [0-1 Mb – black, 0-500 Kb-green, 0-250 Kb- red and 0-100 Kb-blue].*

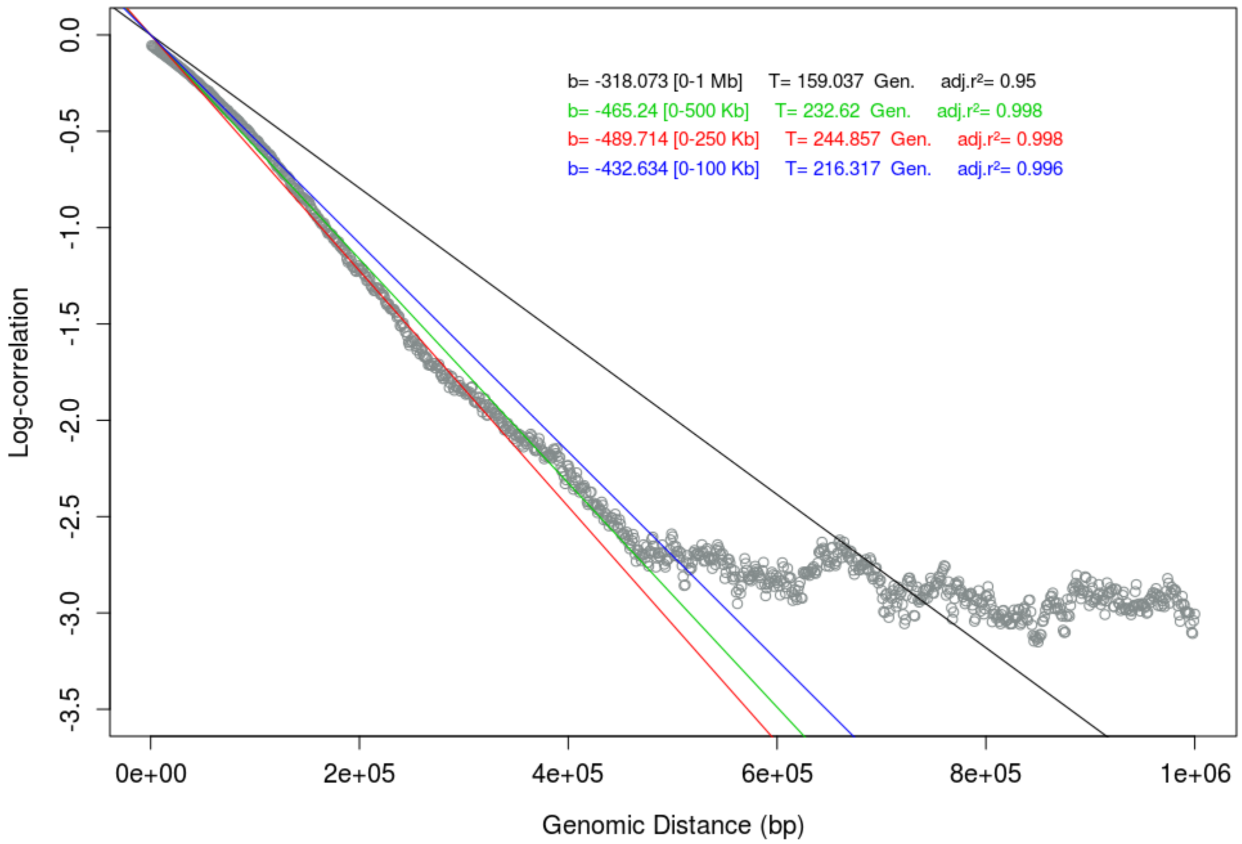

Supplementary Figure 8. *Persistency of Linkage Disequilibrium (LD) measured as the log-correlation between estimates of LD between populations of Avileña Negra-Ibérica -ANI- and Morucha -Mo- and estimates of the slope of the regression with respect to the genomic distance for different ranges [0-1 Mb – black, 0-500 Kb-green, 0-250 Kb- red and 0-100 Kb-blue].*

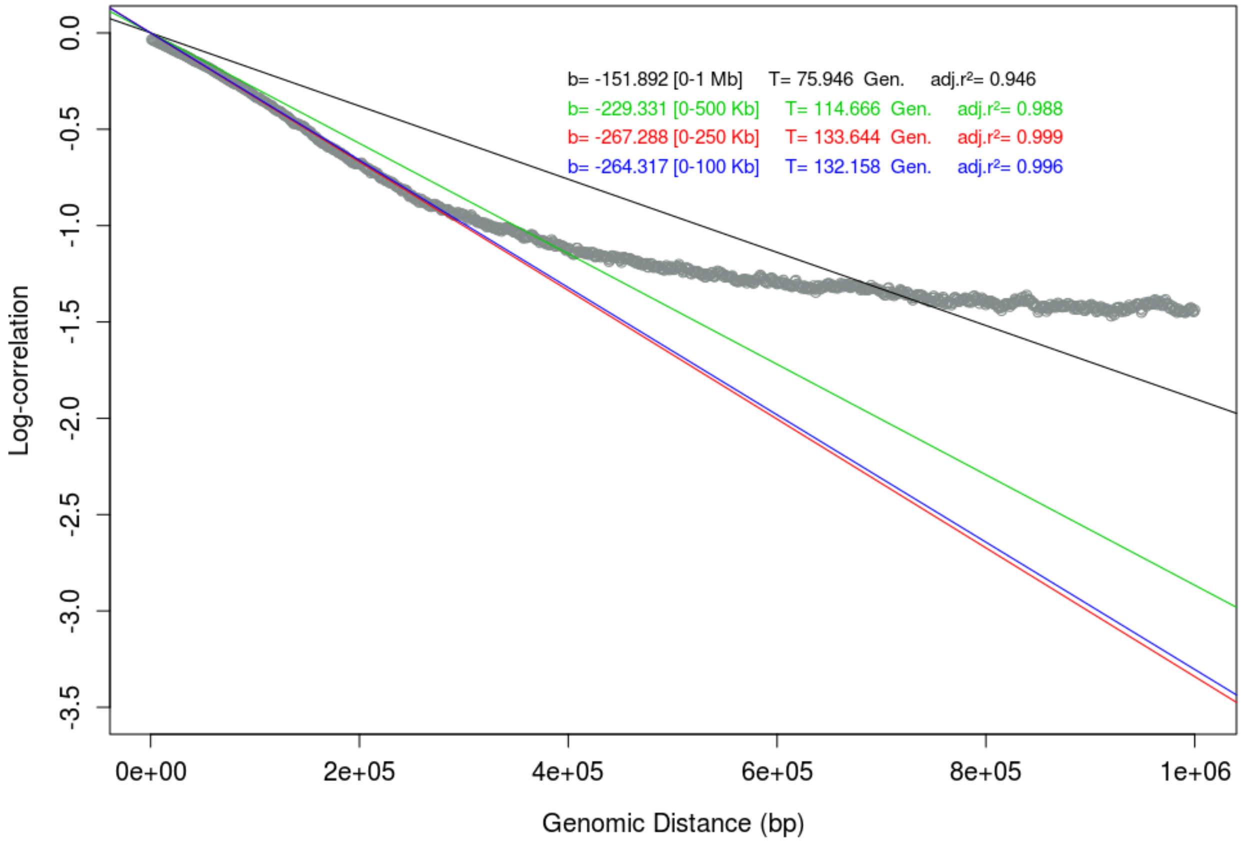

Supplementary Figure 9. *Persistency of Linkage Disequilibrium (LD) measured as the log-correlation between estimates of LD between populations of Avileña Negra-Ibérica -ANI- and Pirenaica -Pi- and estimates of the slope of the regression with respect to the genomic distance for different ranges [0-1 Mb – black, 0-500 Kb-green, 0-250 Kb- red and 0-100 Kb-blue].*

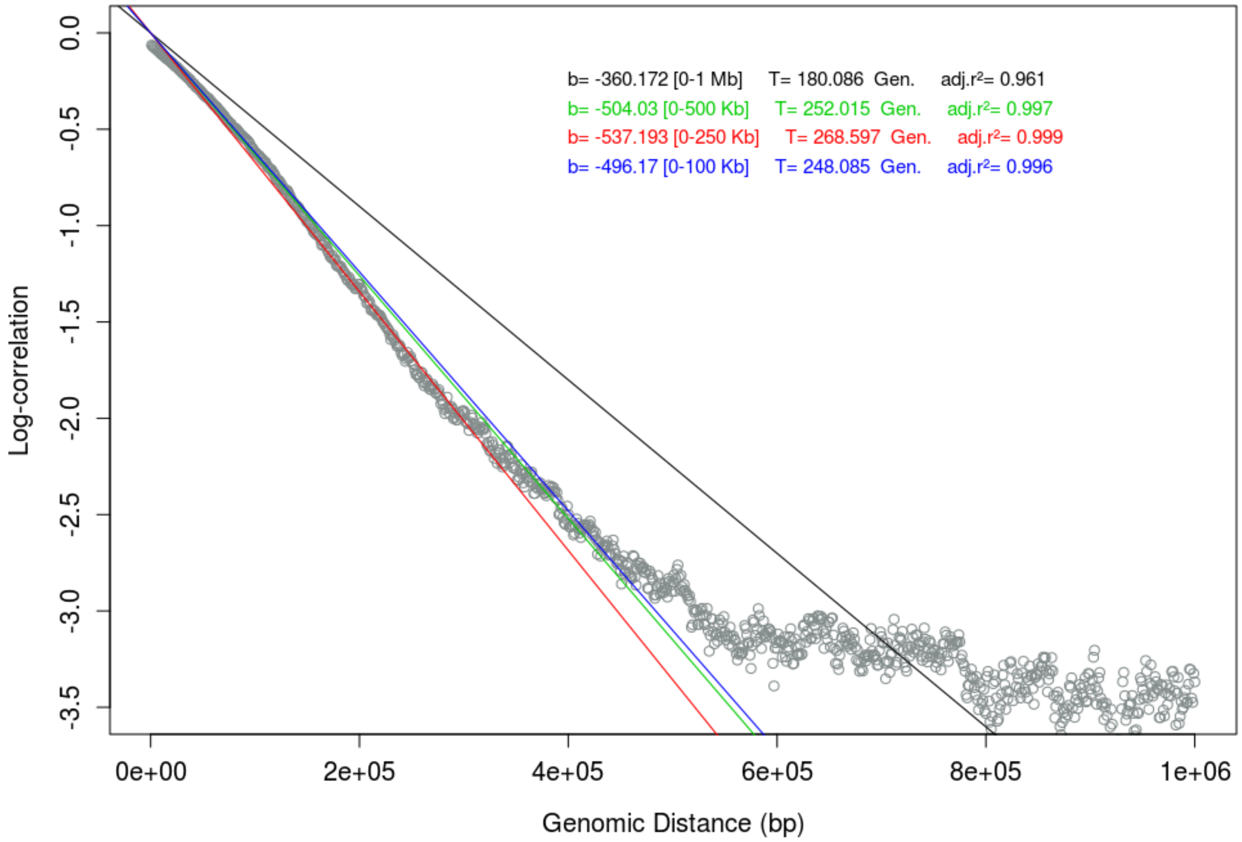

Supplementary Figure 10. *Persistency of Linkage Disequilibrium (LD) measured as the log-correlation between estimates of LD between populations of Avileña Negra-Ibérica -ANI- and Retinta -Re- and estimates of the slope of the regression with respect to the genomic distance for different ranges [0-1 Mb – black, 0-500 Kb-green, 0-250 Kb- red and 0-100 Kb-blue].*

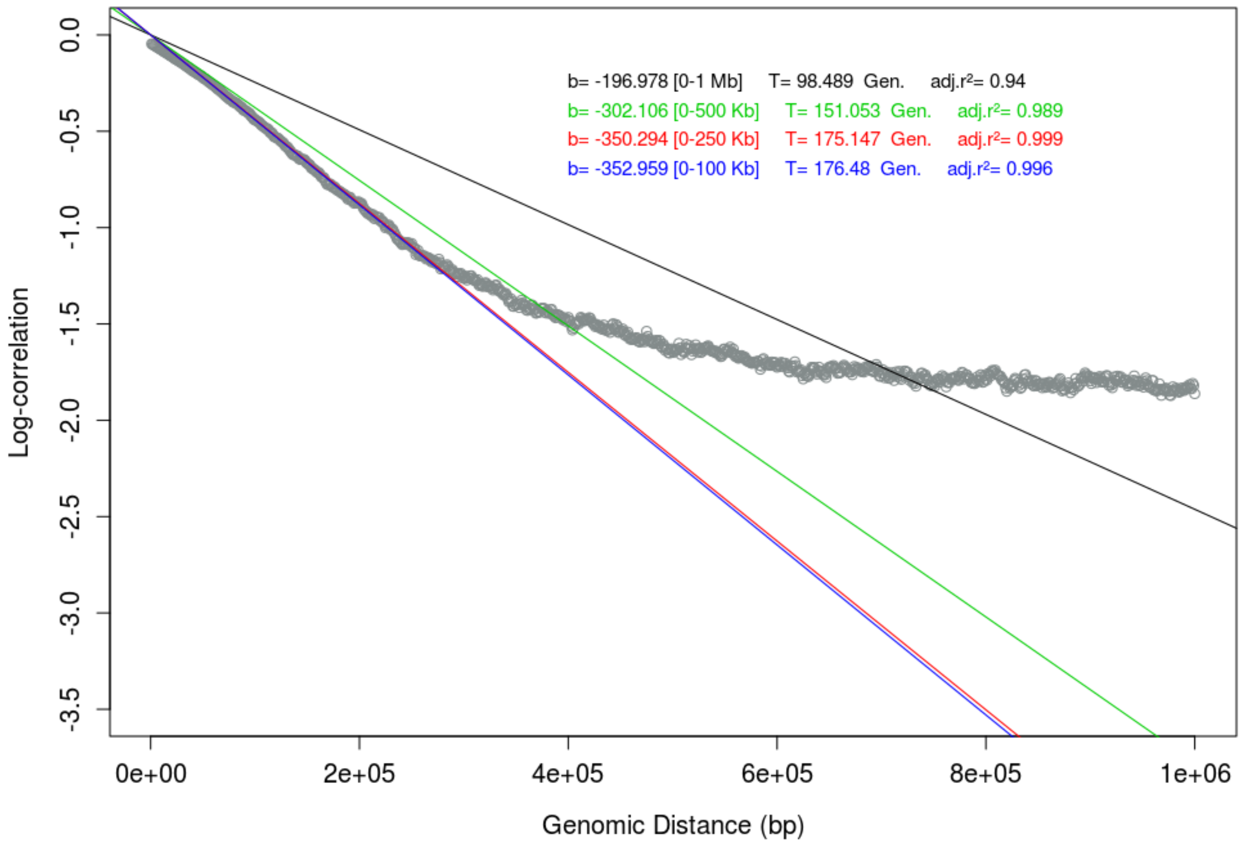

Supplementary Figure 11. *Persistency of Linkage Disequilibrium (LD) measured as the log-correlation between estimates of LD between populations of Avileña Negra-Ibérica -ANI- and Rubia Gallega -RG- and estimates of the slope of the regression with respect to the genomic distance for different ranges [0-1 Mb – black, 0-500 Kb-green, 0-250 Kb- red and 0-100 Kb-blue].*

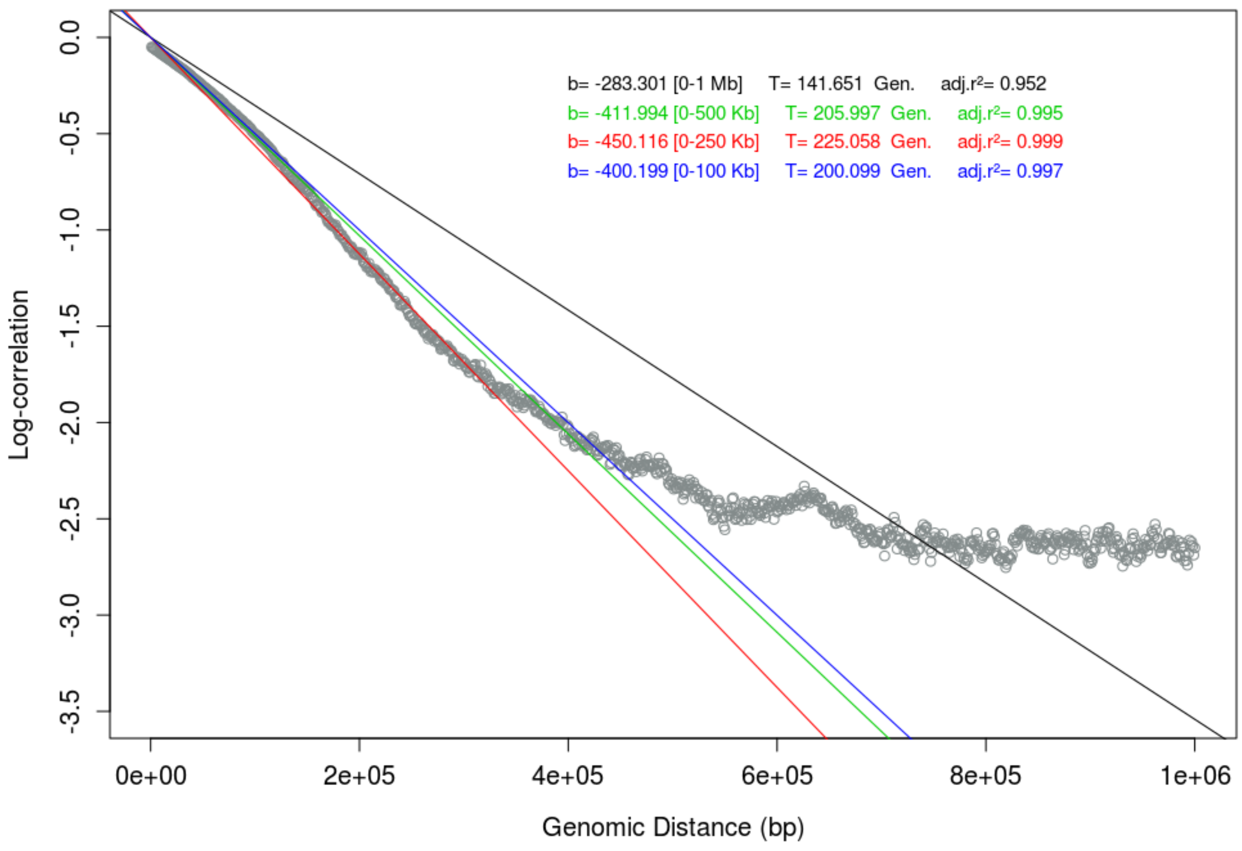

Supplementary Figure 12. *Persistency of Linkage Disequilibrium (LD) measured as the log-correlation between estimates of LD between populations of Bruna dels Pirineus -BP- and Morucha -Mo- and estimates of the slope of the regression with respect to the genomic distance for different ranges [0-1 Mb – black, 0-500 Kb-green, 0-250 Kb- red and 0-100 Kb-blue]*.

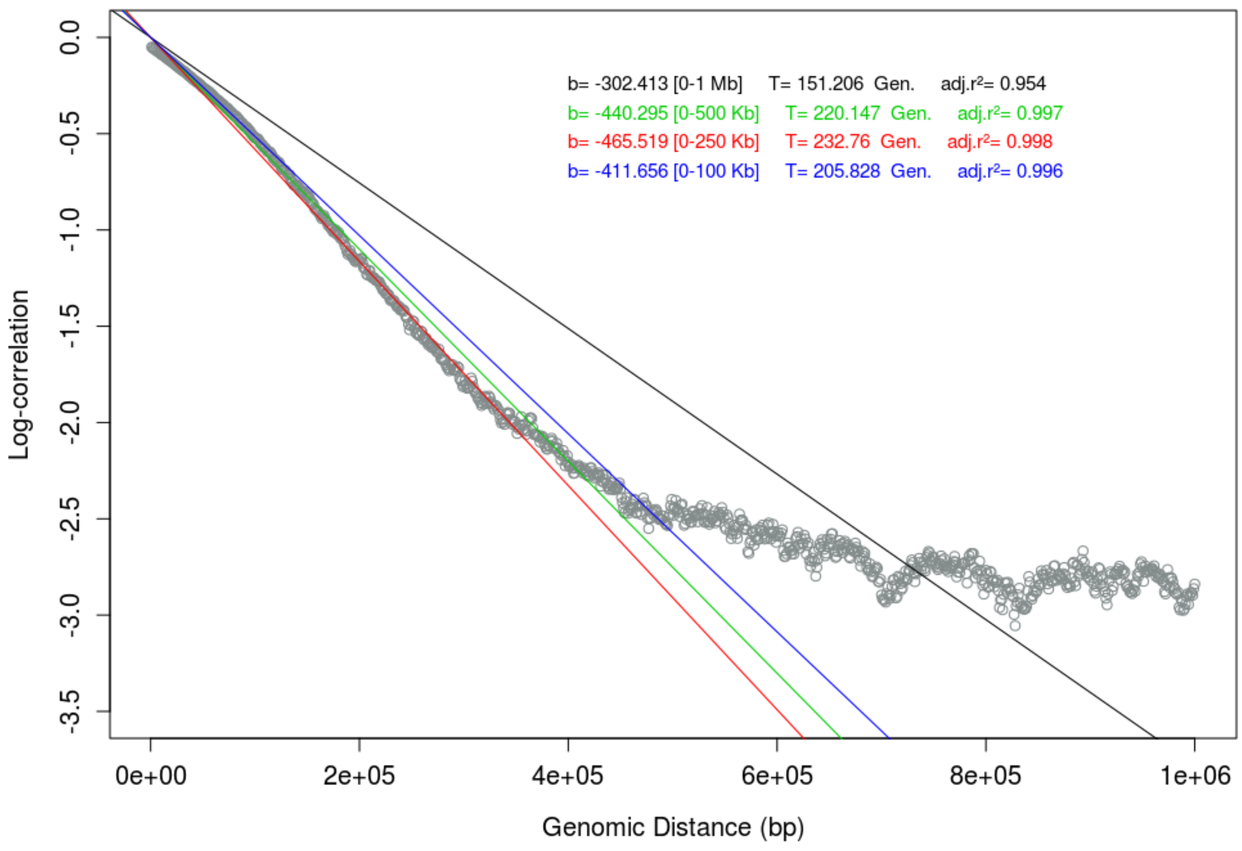

Supplementary Figure 13. *Persistency of Linkage Disequilibrium (LD) measured as the log-correlation between estimates of LD between populations of Bruna dels Pirineus -BP- and Pirenaica -Pi- and estimates of the slope of the regression with respect to the genomic distance for different ranges [0-1 Mb – black, 0-500 Kb-green, 0-250 Kb- red and 0-100 Kb-blue].*

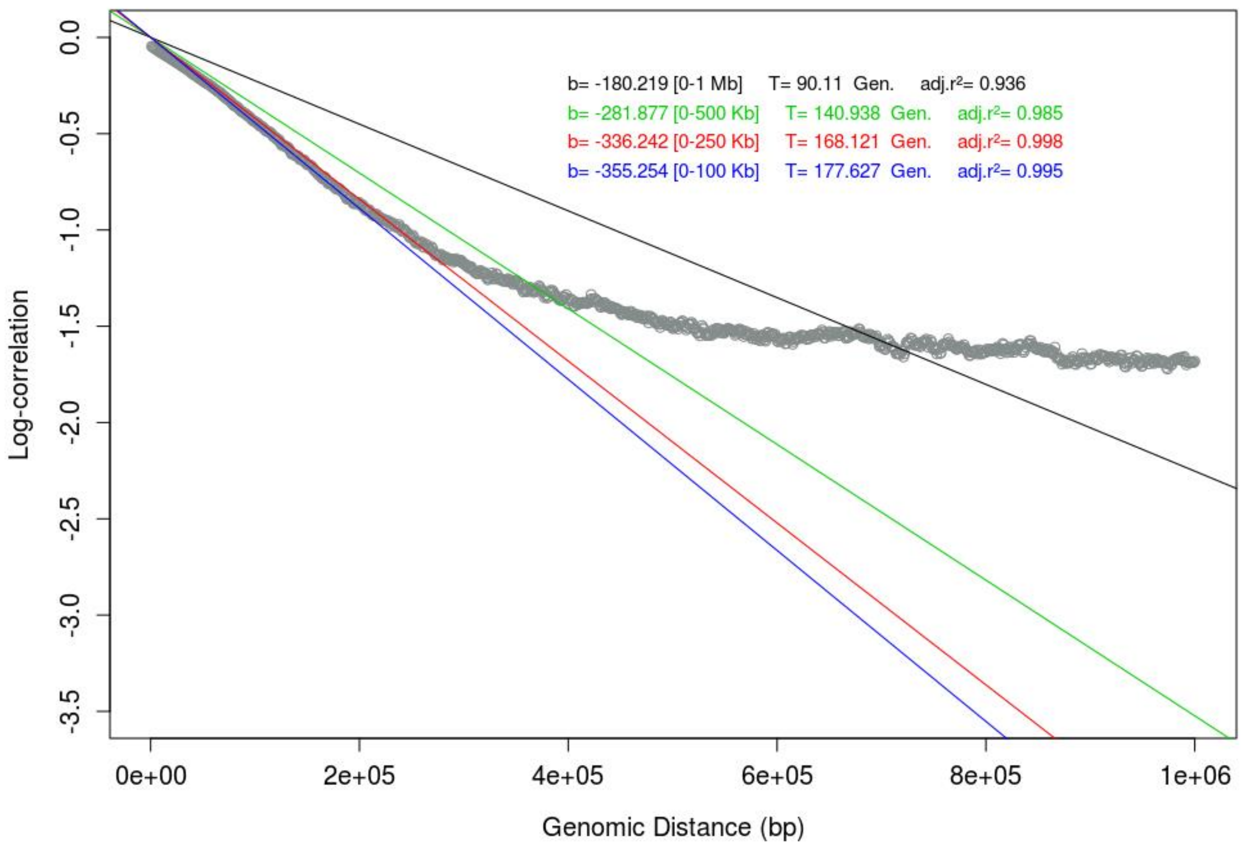

Supplementary Figure 14. *Persistency of Linkage Disequilibrium (LD) measured as the log-correlation between estimates of LD between populations of Bruna dels Pirineus -BP- and Retinta -Re- and estimates of the slope of the regression with respect to the genomic distance for different ranges [0-1 Mb – black, 0-500 Kb-green, 0-250 Kb- red and 0-100 Kb-blue].*

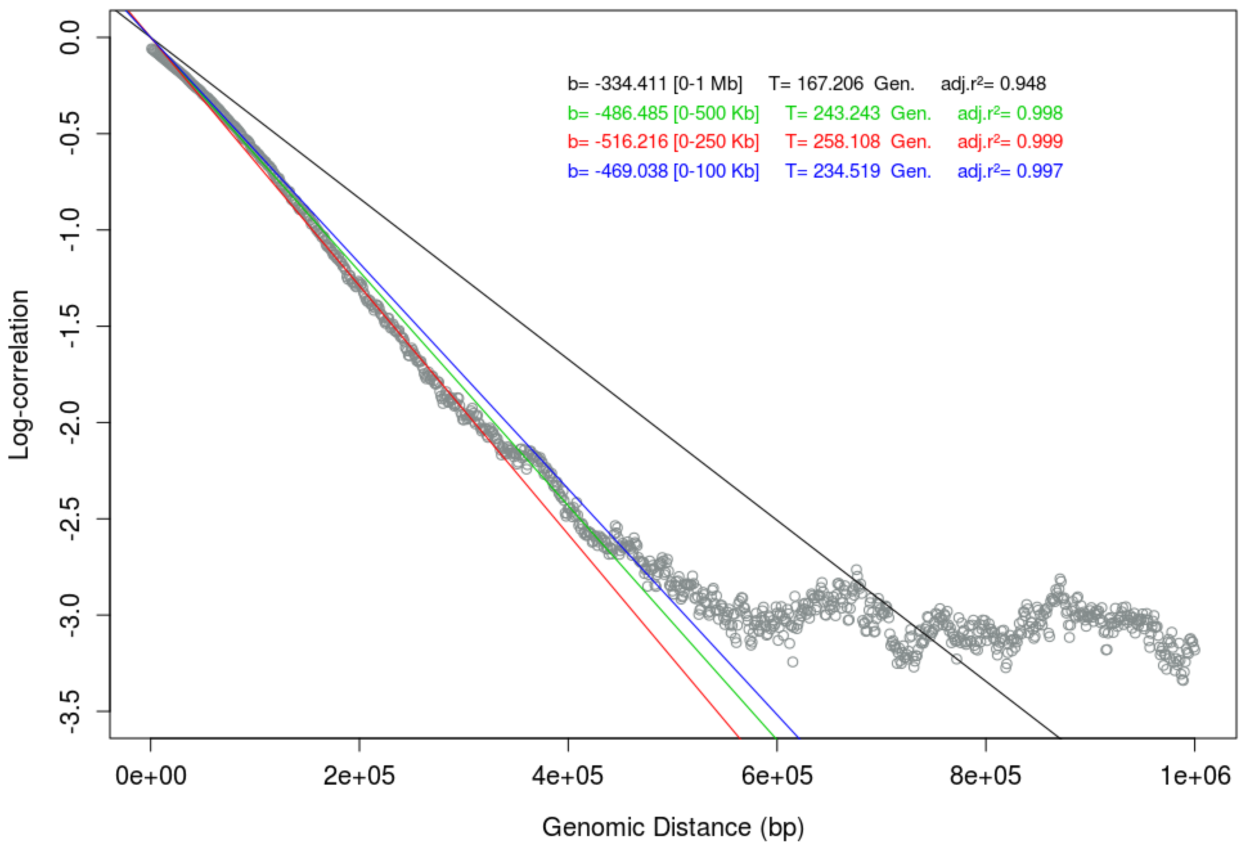

Supplementary Figure 15. *Persistency of Linkage Disequilibrium (LD) measured as the log-correlation between estimates of LD between populations of Bruna dels Pirineus -BP- and Rubia Gallega -RG- and estimates of the slope of the regression with respect to the genomic distance for different ranges [0-1 Mb – black, 0-500 Kb-green, 0-250 Kb- red and 0-100 Kb-blue].*

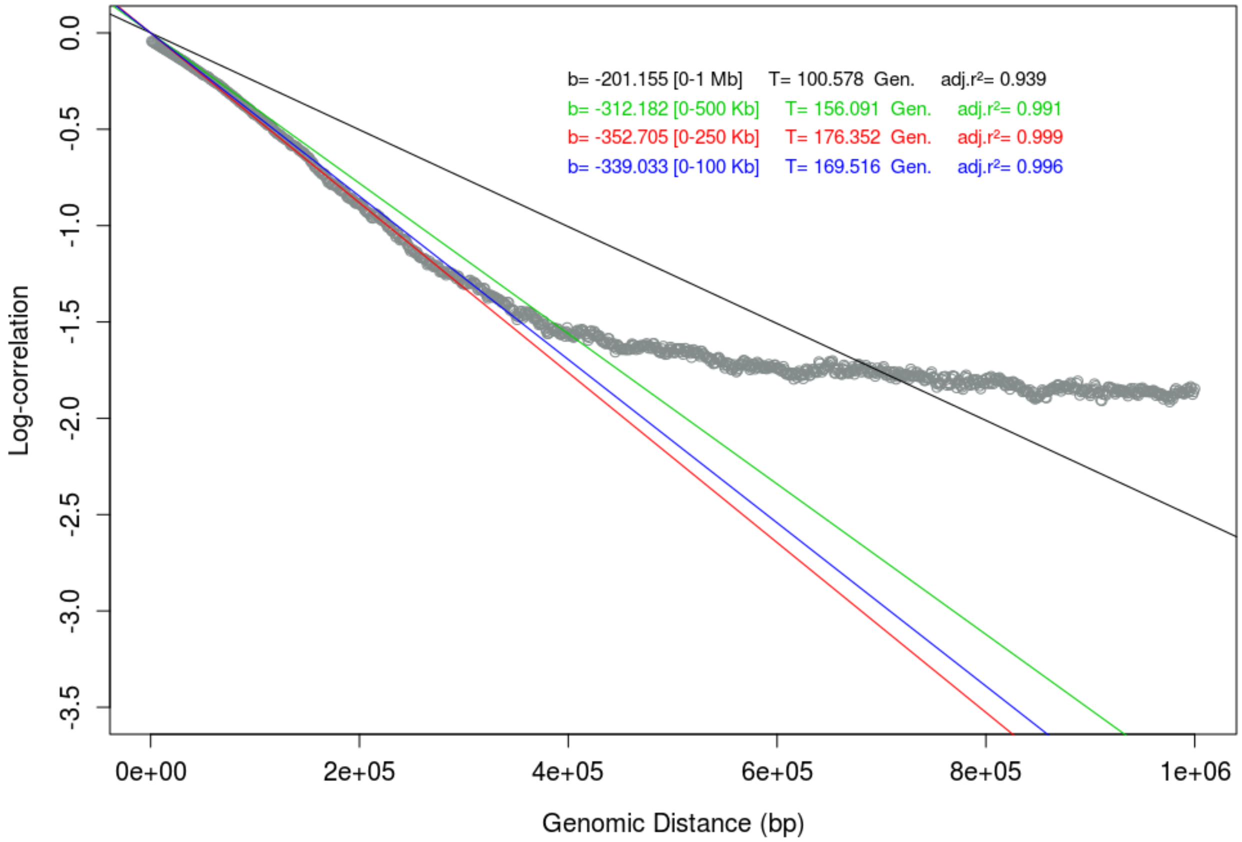

Supplementary Figure 16. *Persistency of Linkage Disequilibrium (LD) measured as the log-correlation between estimates of LD between populations of Morucha -Mo- and Pirenaica -Pi- and estimates of the slope of the regression with respect to the genomic distance for different ranges [0-1 Mb – black, 0-500 Kb-green, 0-250 Kb- red and 0-100 Kb-blue].*

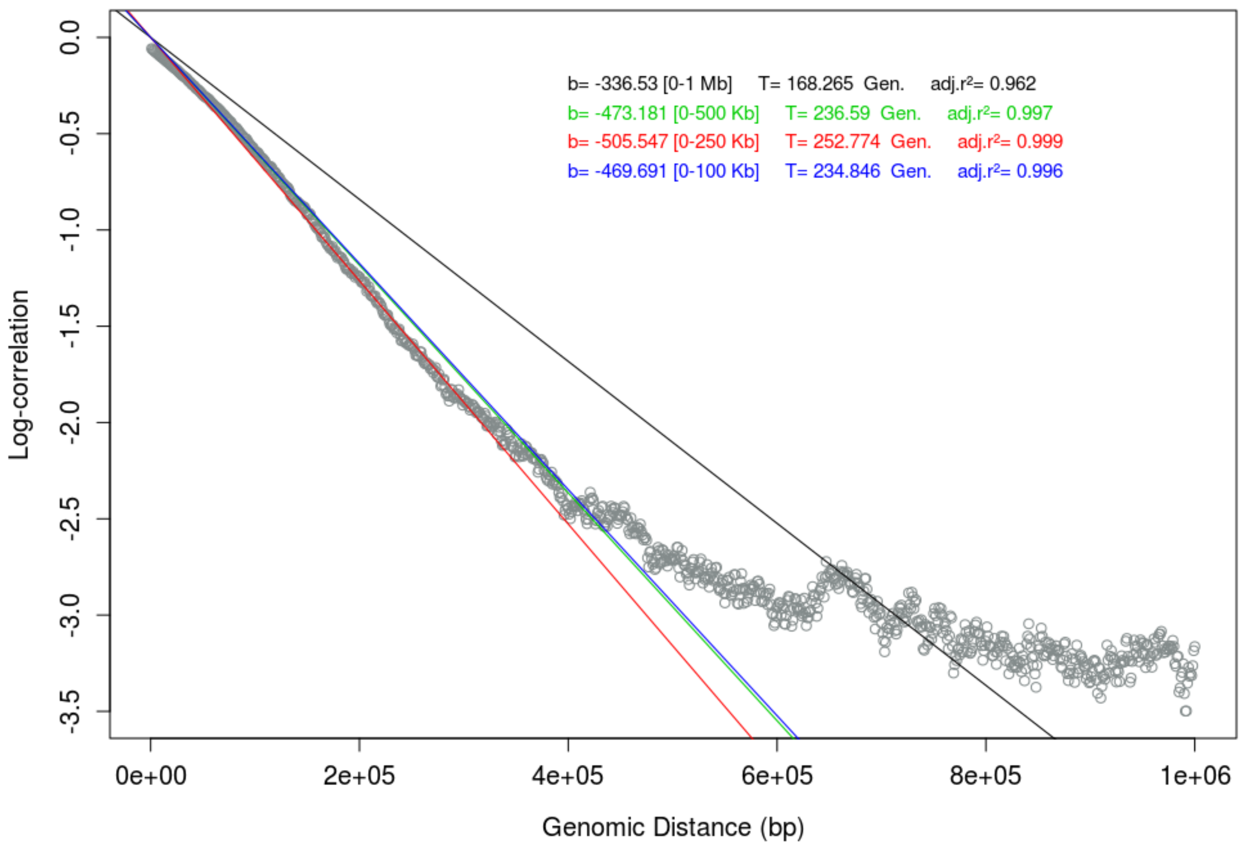

Supplementary Figure 17. *Persistency of Linkage Disequilibrium (LD) measured as the log-correlation between estimates of LD between populations of Morucha -Mo- and Retinta -Re- and estimates of the slope of the regression with respect to the genomic distance for different ranges [0-1 Mb – black, 0-500 Kb-green, 0-250 Kb- red and 0-100 Kb-blue].*

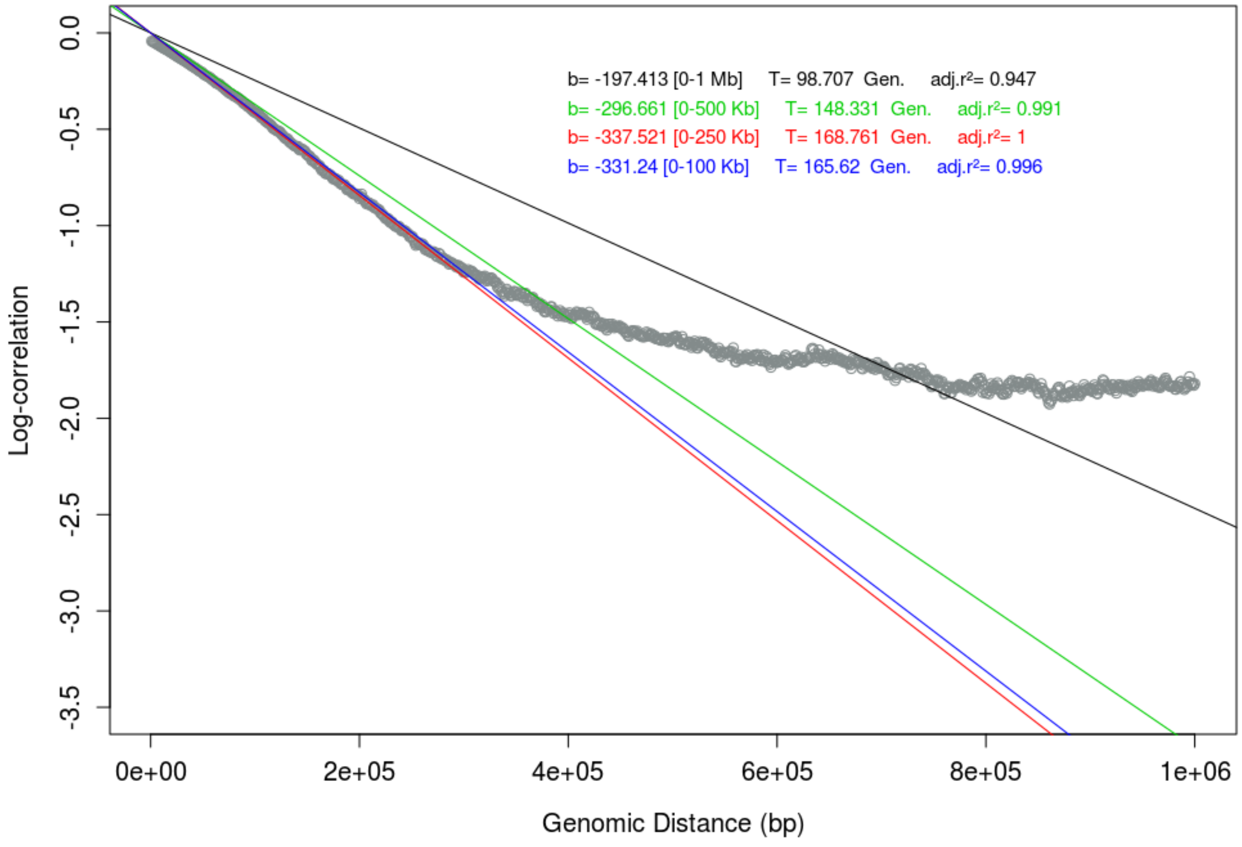

Supplementary Figure 18. *Persistency of Linkage Disequilibrium (LD) measured as the log-correlation between estimates of LD between populations of Morucha -Mo- and Rubia Gallega -RG- and estimates of the slope of the regression with respect to the genomic distance for different ranges [0-1 Mb – black, 0-500 Kb-green, 0-250 Kb- red and 0-100 Kb-blue].*

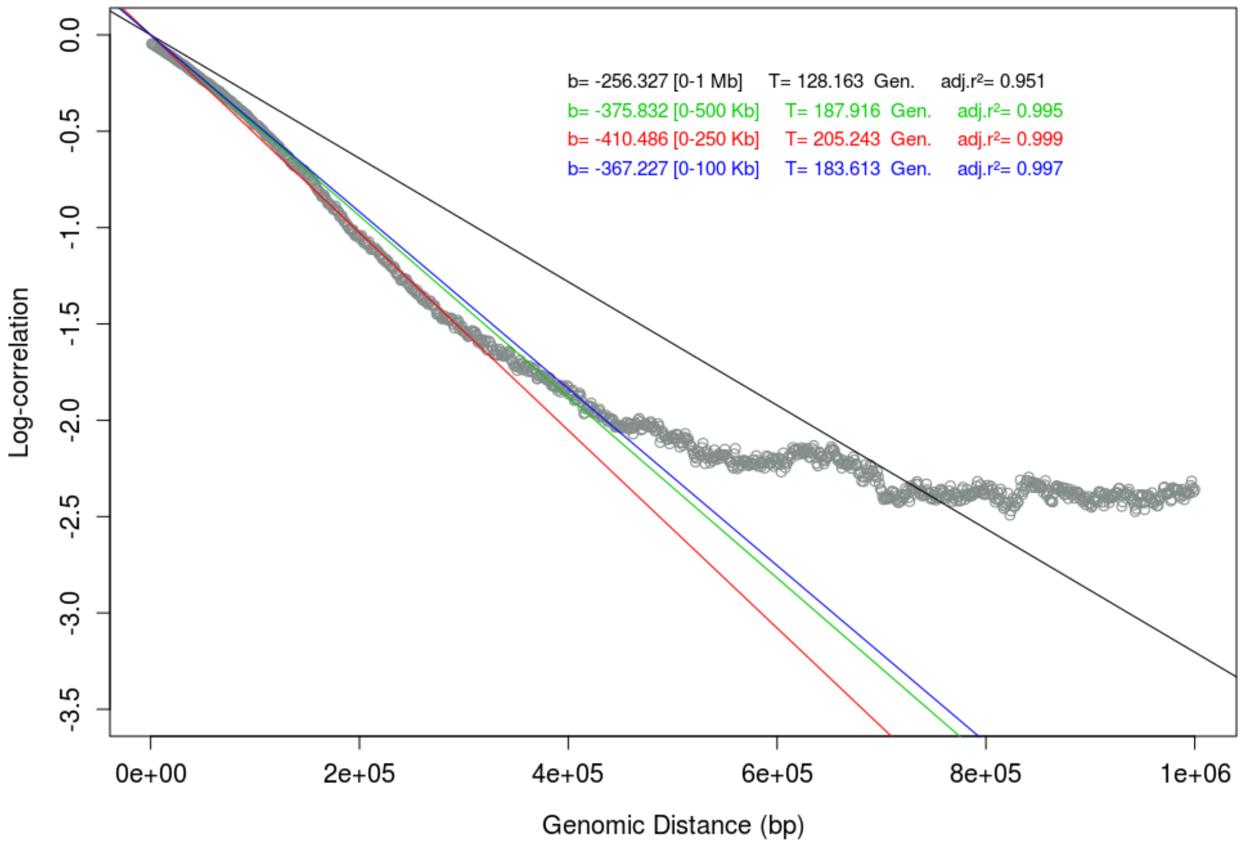

Supplementary Figure 19. *Persistency of Linkage Disequilibrium (LD) measured as the log-correlation between estimates of LD between populations of Pirenaica -Pi- and Retinta -Re- and estimates of the slope of the regression with respect to the genomic distance for different ranges [0-1 Mb – black, 0-500 Kb-green, 0-250 Kb- red and 0-100 Kb-blue].*

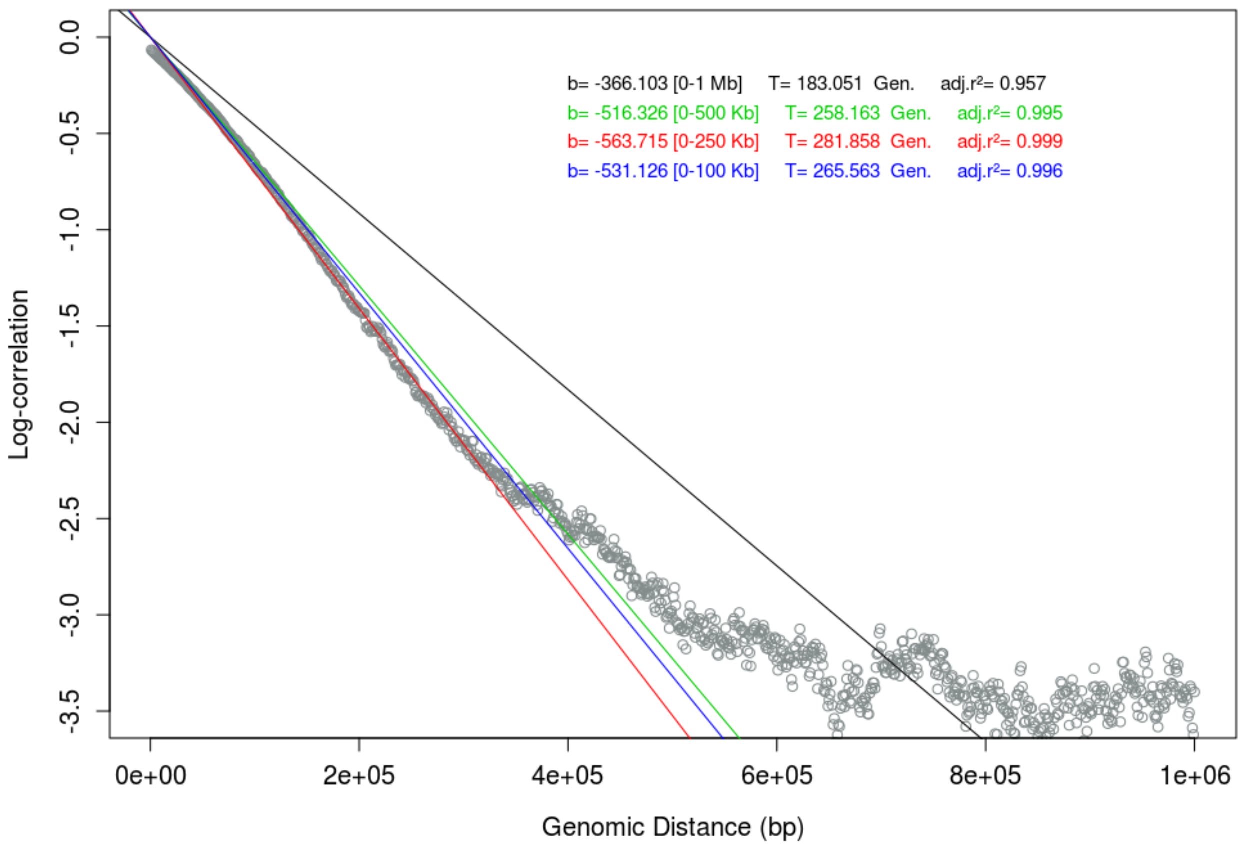

Supplementary Figure 20. *Persistency of Linkage Disequilibrium (LD) measured as the log-correlation between estimates of LD between populations of Rubia Gallega -RG- and Retinta -Re- and estimates of the slope of the regression with respect to the genomic distance for different ranges [0-1 Mb – black, 0-500 Kb-green, 0-250 Kb- red and 0-100 Kb-blue]*.

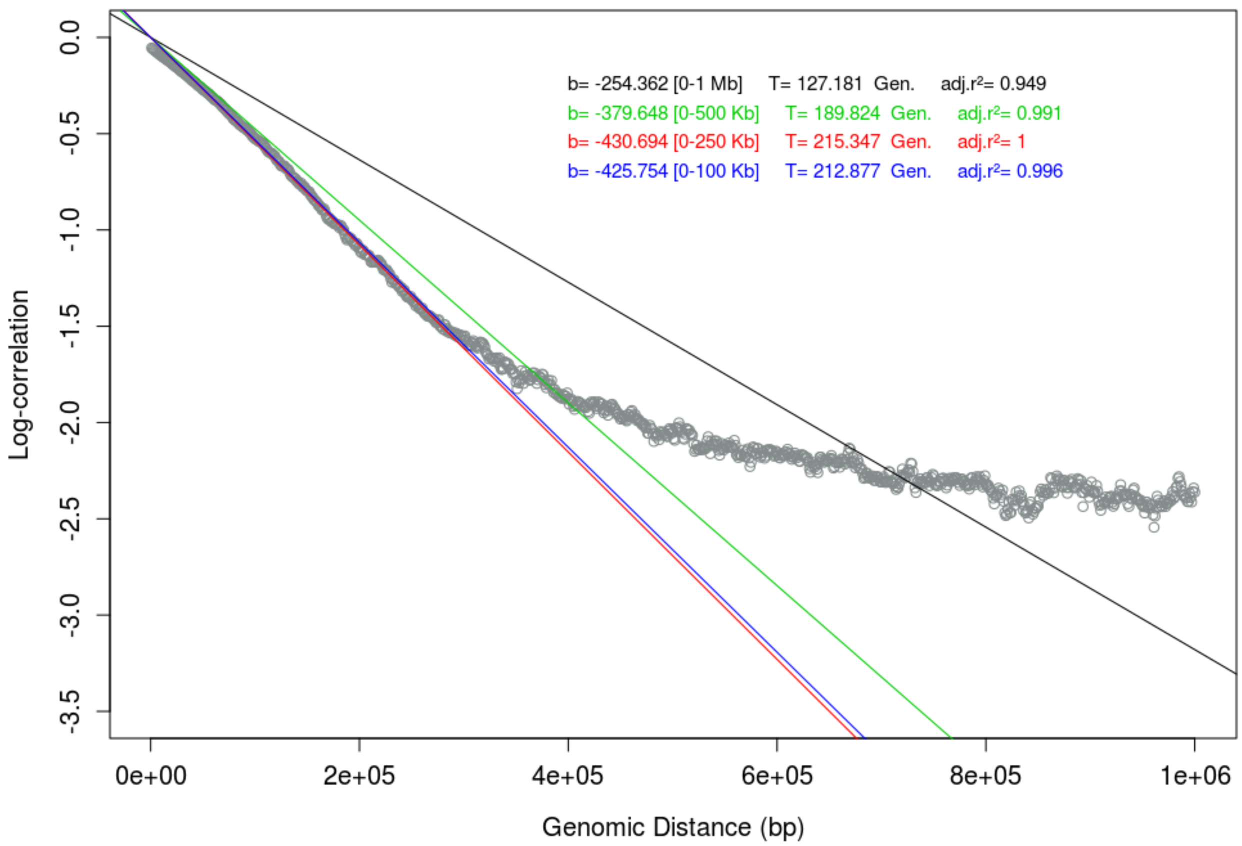

Supplementary Figure 21. *Estimates of the recombination rate (c) in bins of 1 Mb between Asturiana de los Valles -AV- and Avileña Negra-Ibérica -ANI- throughout the autosomal genome.*

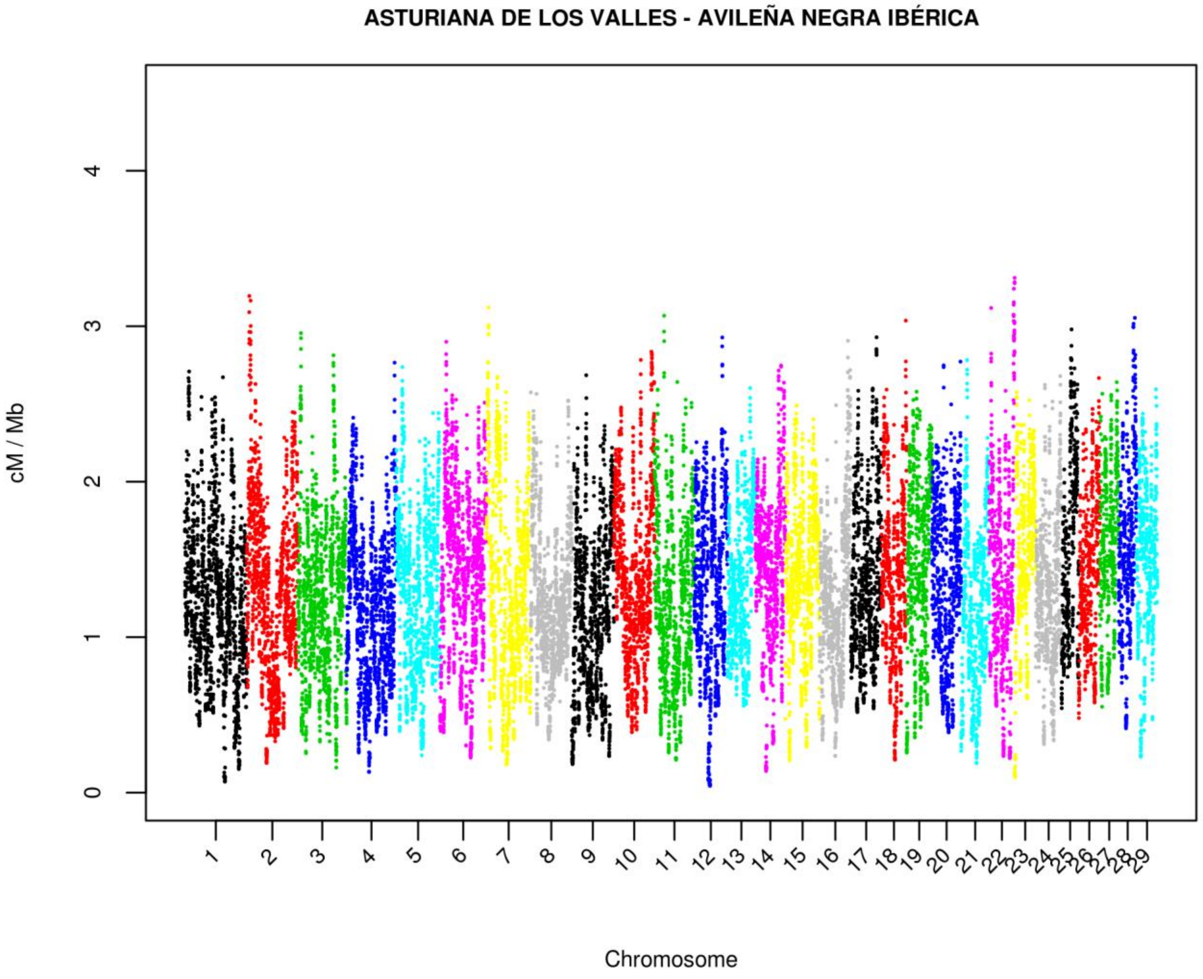

Supplementary Figure 22. *Estimates of the recombination rate (c) in bins of 1 Mb between Asturiana de los Valles -AV- and Avileña Negra-Ibérica -ANI- throughout the autosomal genome.*

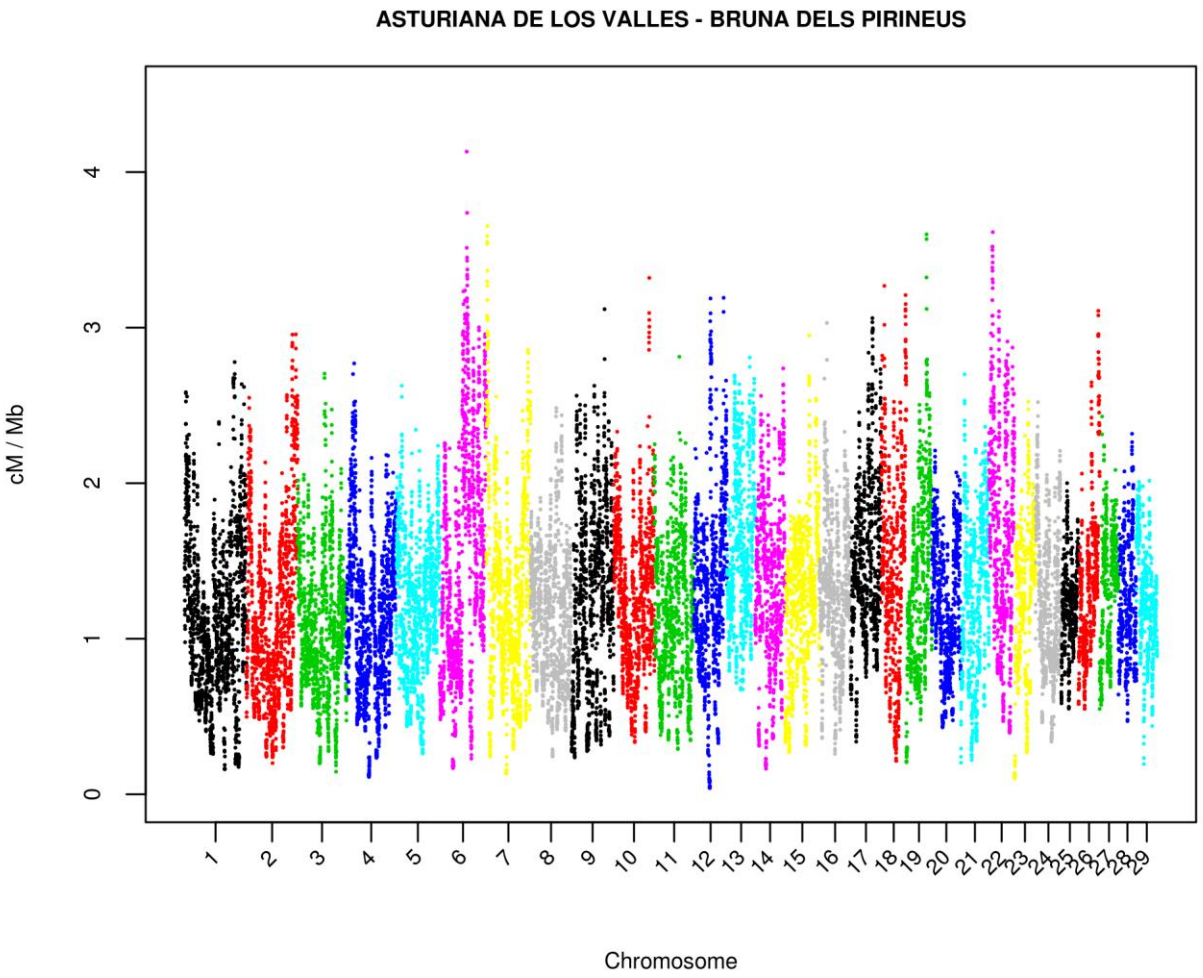

Supplementary Figure 23. *Estimates of the recombination rate (c) in bins of 1 Mb between Asturiana de los Valles -AV- and Morucha -Mo- throughout the autosomal genome.*

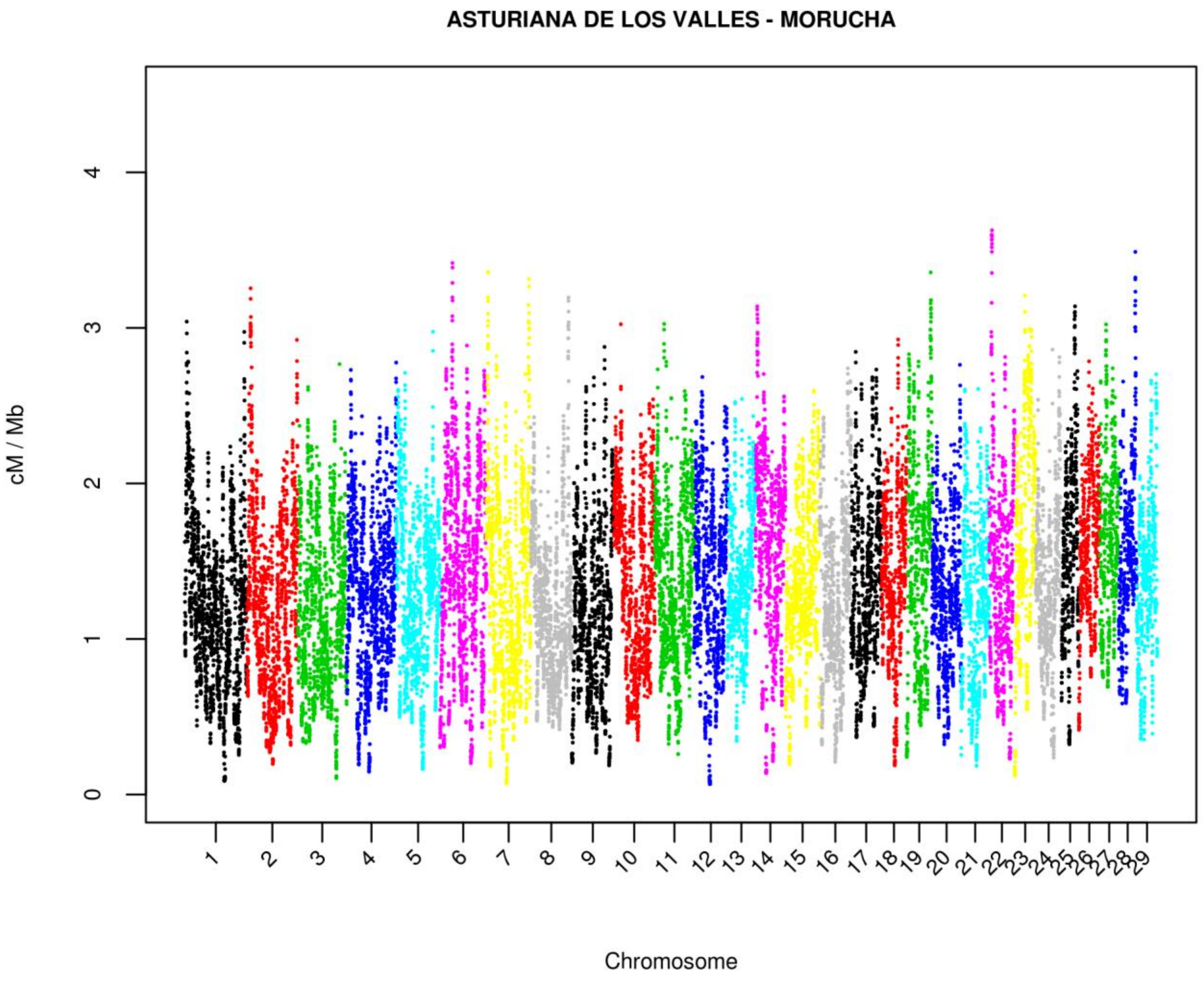

Supplementary Figure 24. *Estimates of the recombination rate (c) in bins of 1 Mb between Asturiana de los Valles -AV- and Pirenaica -Pi- throughout the autosomal genome.*

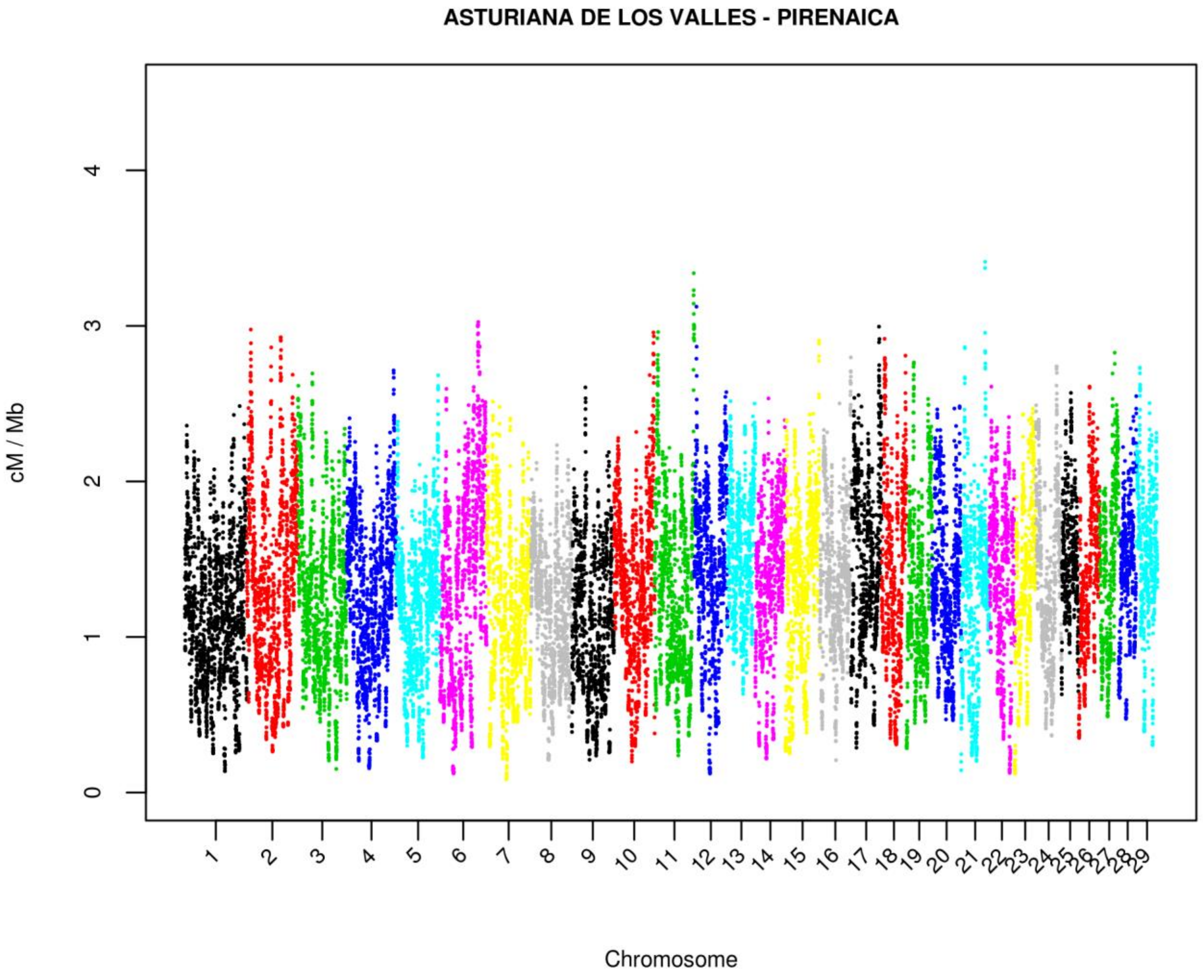

Supplementary Figure 25. *Estimates of the recombination rate (c) in bins of 1 Mb between Asturiana de los Valles -AV- and Retinta -Re- throughout the autosomal genome.*

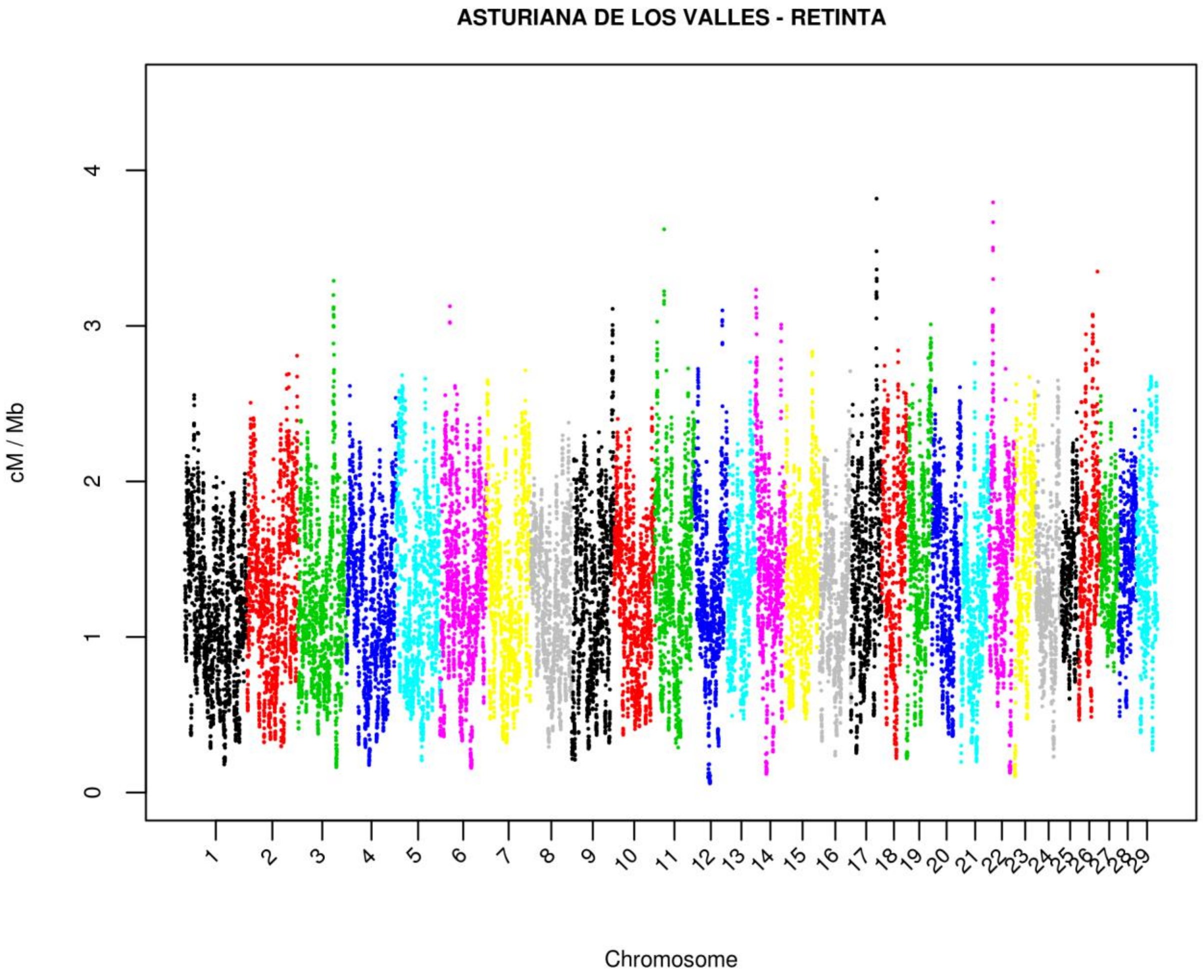

Supplementary Figure 26. *Estimates of the recombination rate (c) in bins of 1 Mb between Asturiana de los Valles -AV- and Rubia Gallega – RG- throughout the autosomal genome.*

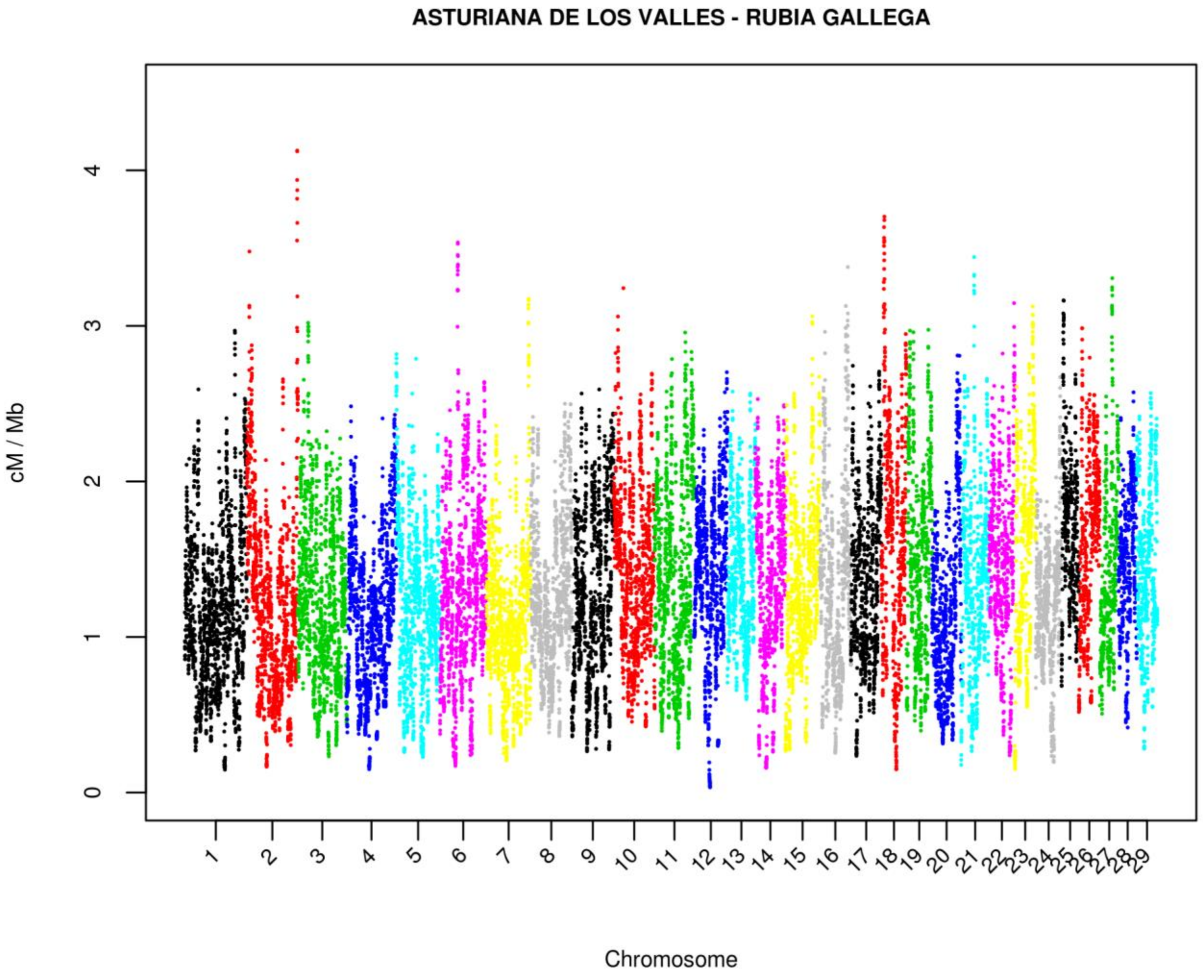

Supplementary Figure 27. *Estimates of the recombination rate (c) in bins of 1 Mb between Avileña Negra-Ibérica -ANI- and Bruna dels Pirineus – BP- throughout the autosomal genome.*

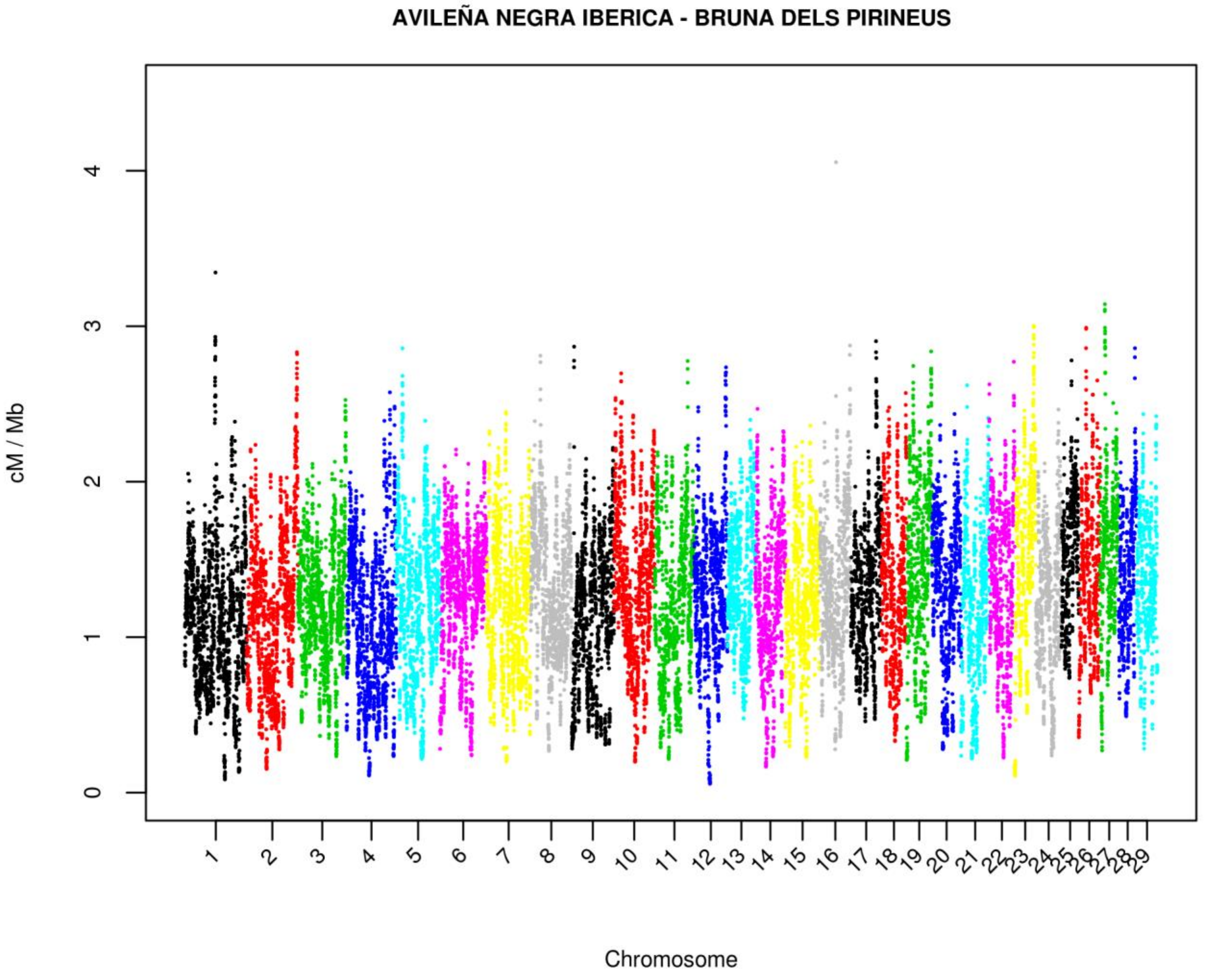

Supplementary Figure 28. *Estimates of the recombination rate (c) in bins of 1 Mb between Avileña Negra-Ibérica -ANI- and Morucha – Mo- throughout the autosomal genome.*

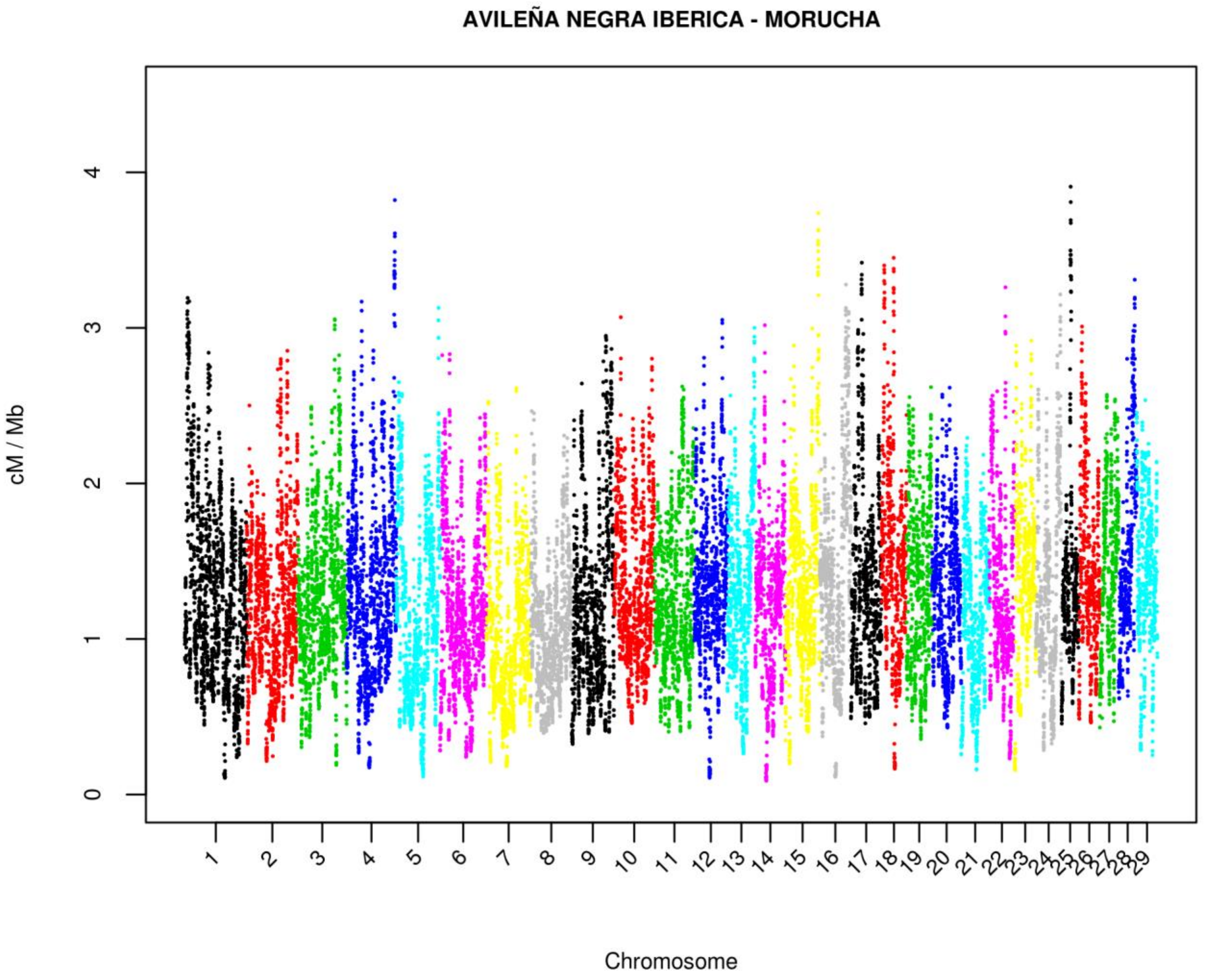

Supplementary Figure 29. *Estimates of the recombination rate (c) in bins of 1 Mb between Avileña Negra-Ibérica -ANI- and Pirenaica – Pi- throughout the autosomal genome.*

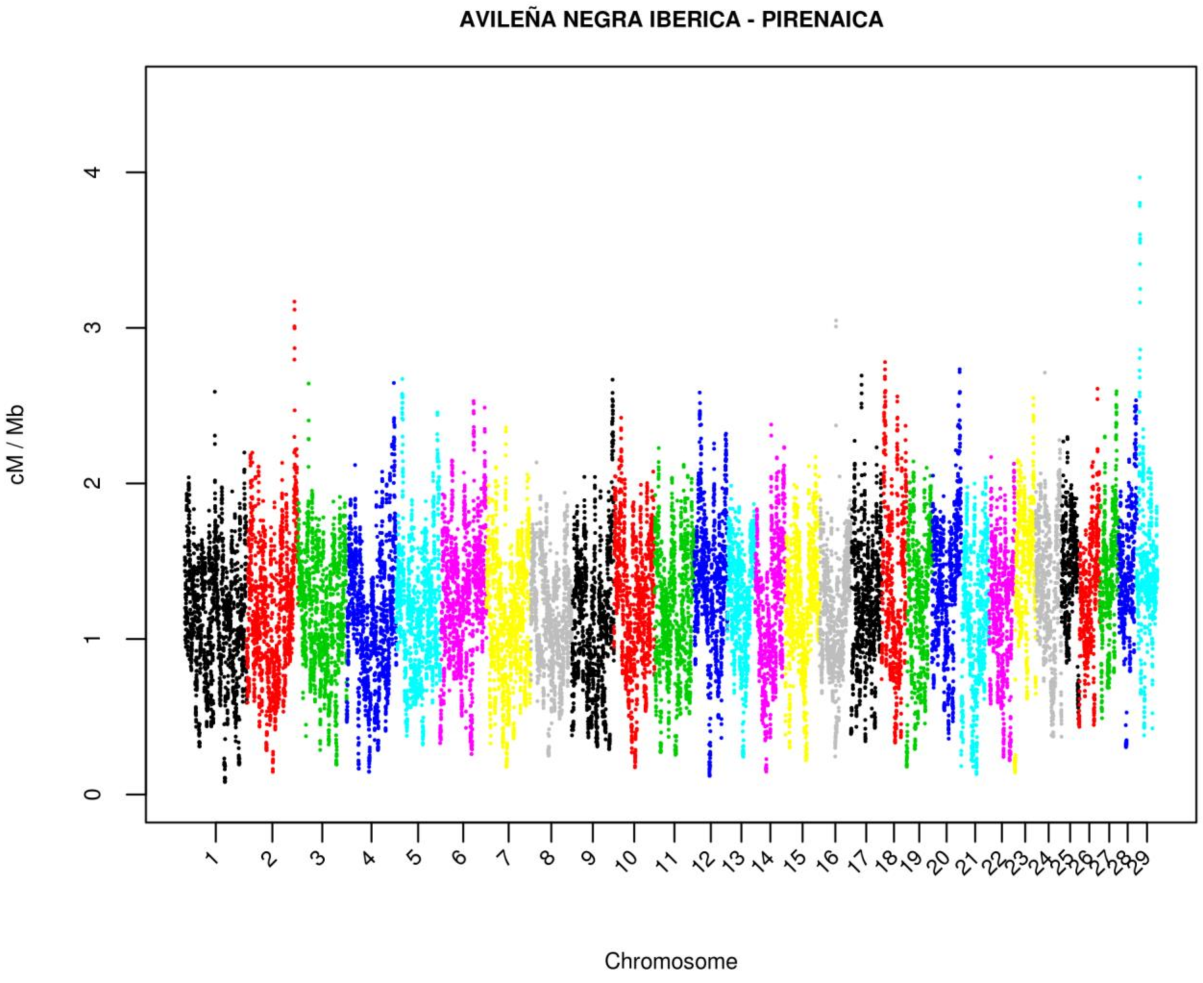

Supplementary Figure 30. *Estimates of the recombination rate (c) in bins of 1 Mb between Avileña Negra-Ibérica -ANI- and Retinta – Re- throughout the autosomal genome.*

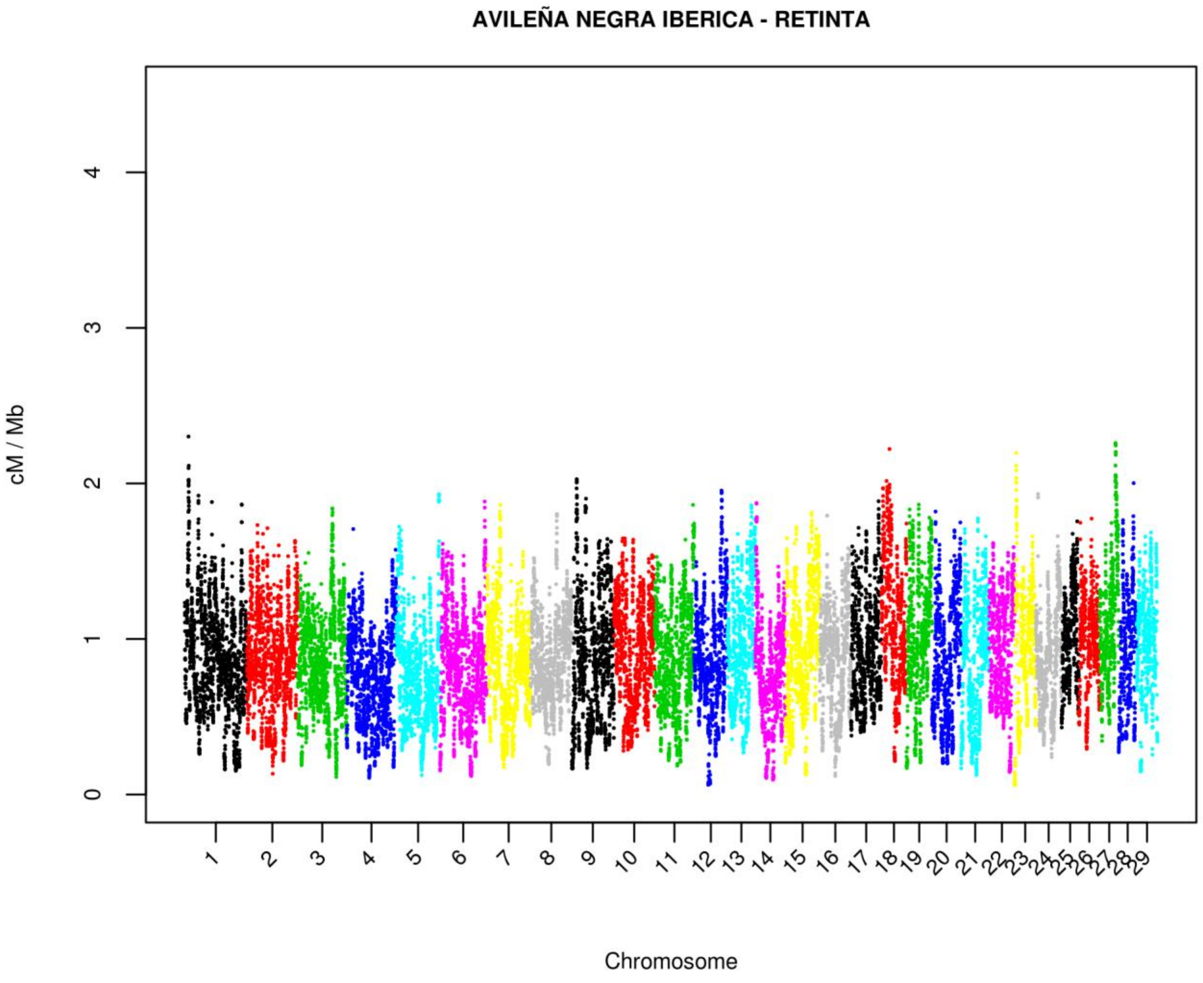

Supplementary Figure 31. *Estimates of the recombination rate (c) in bins of 1 Mb between Avileña Negra-Ibérica -ANI- and Rubia Gallega -RG- throughout the autosomal genome.*

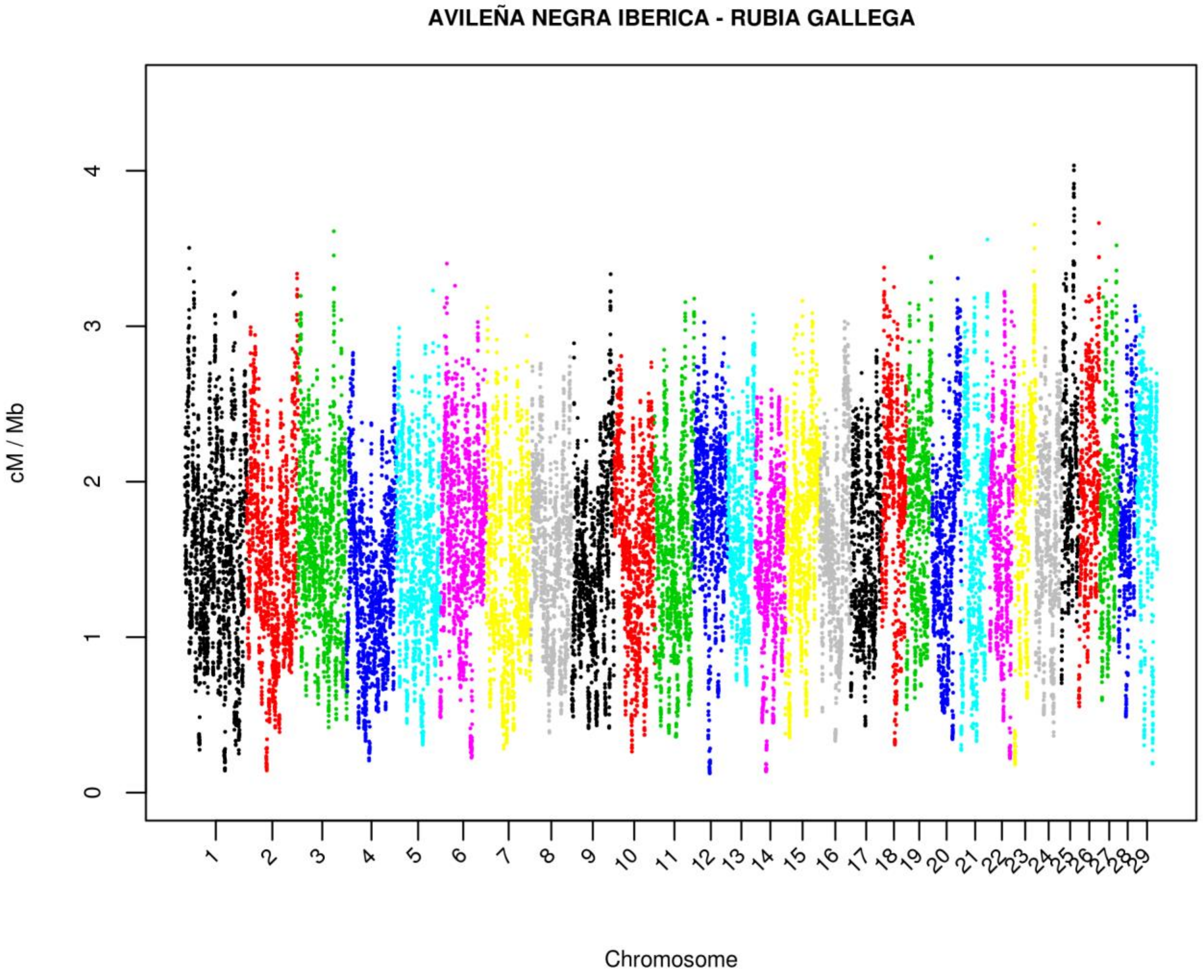

Supplementary Figure 32. *Estimates of the recombination rate (c) in bins of 1 Mb between Bruna dels Pirineus -BP- and Morucha -Mo- throughout the autosomal genome.*

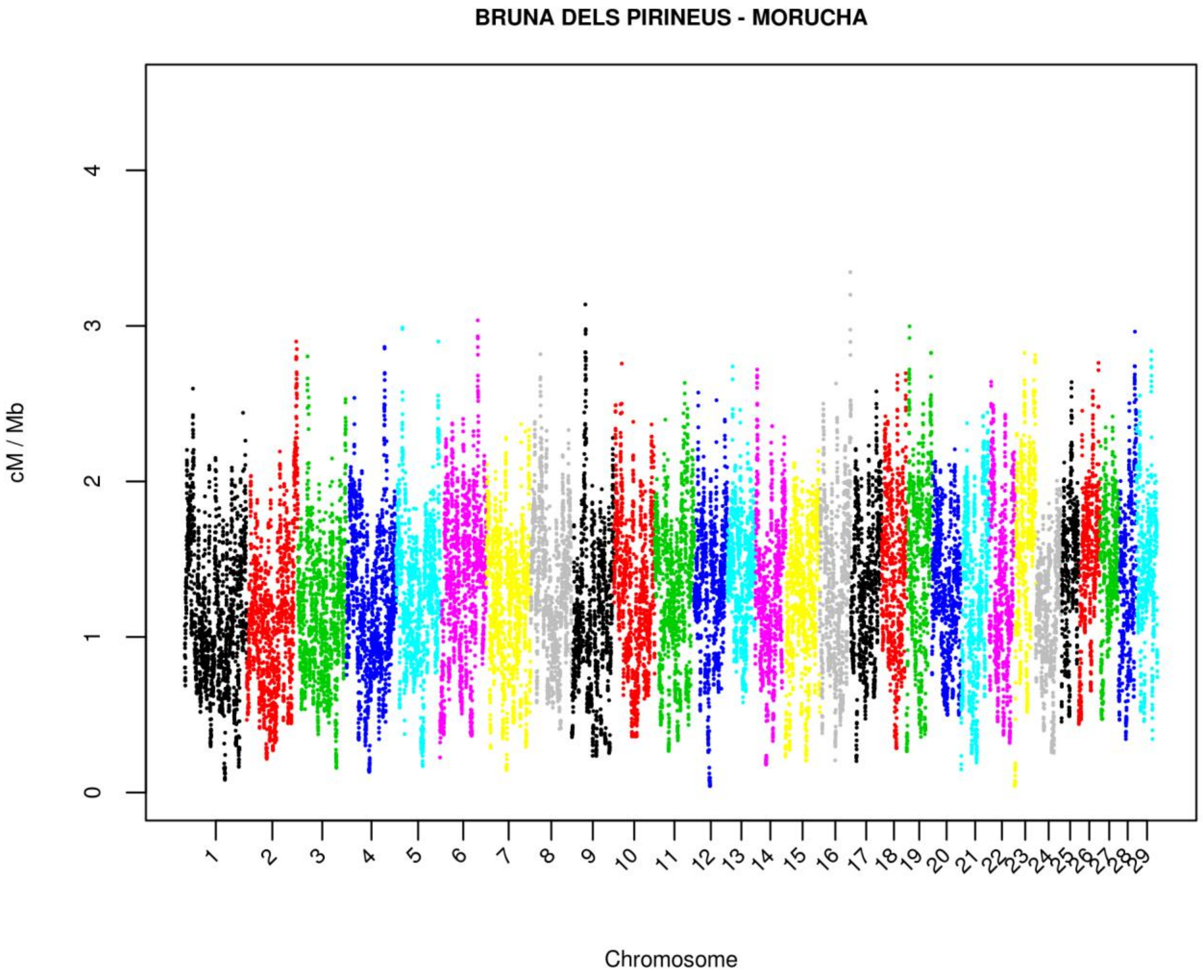

Supplementary Figure 33. *Estimates of the recombination rate (c) in bins of 1 Mb between Bruna dels Pirineus -BP- and Pirenaica -Pi- throughout the autosomal genome.*

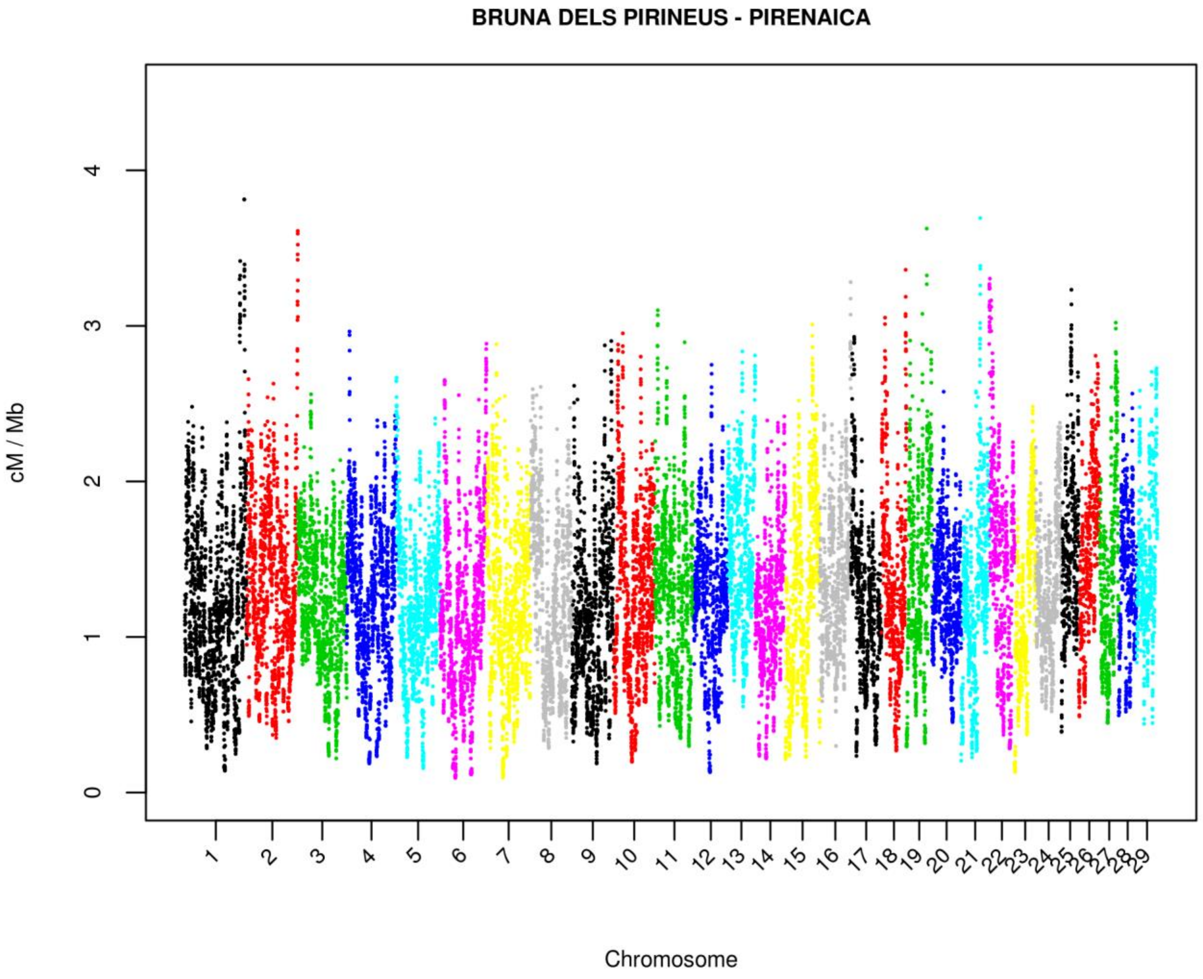

Supplementary Figure 34. *Estimates of the recombination rate (c) in bins of 1 Mb between Bruna dels Pirineus -BP- and Retinta -Re- throughout the autosomal genome.*

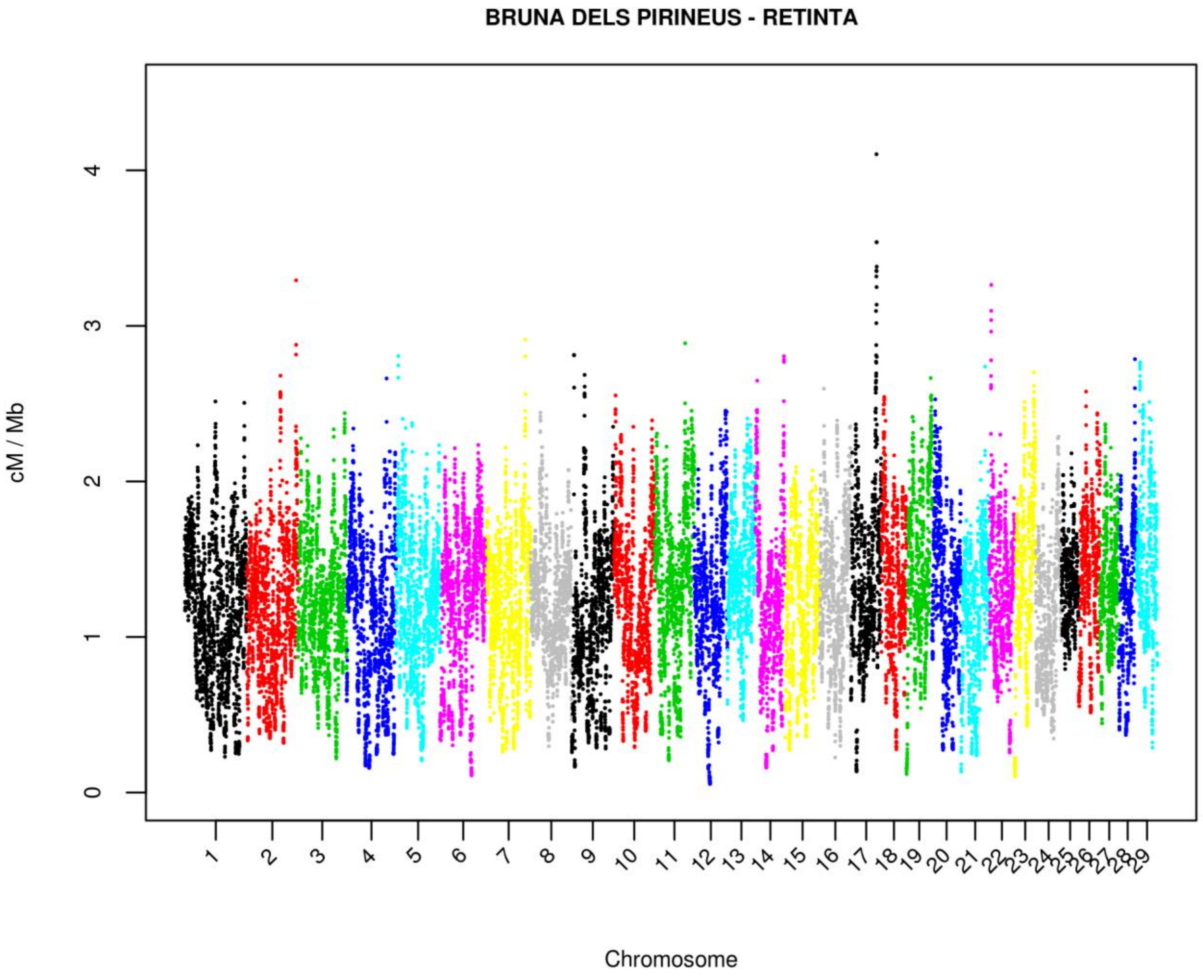

Supplementary Figure 35. *Estimates of the recombination rate (c) in bins of 1 Mb between Bruna dels Pirineus -BP- and Rubia Gallega -RG- throughout the autosomal genome.*

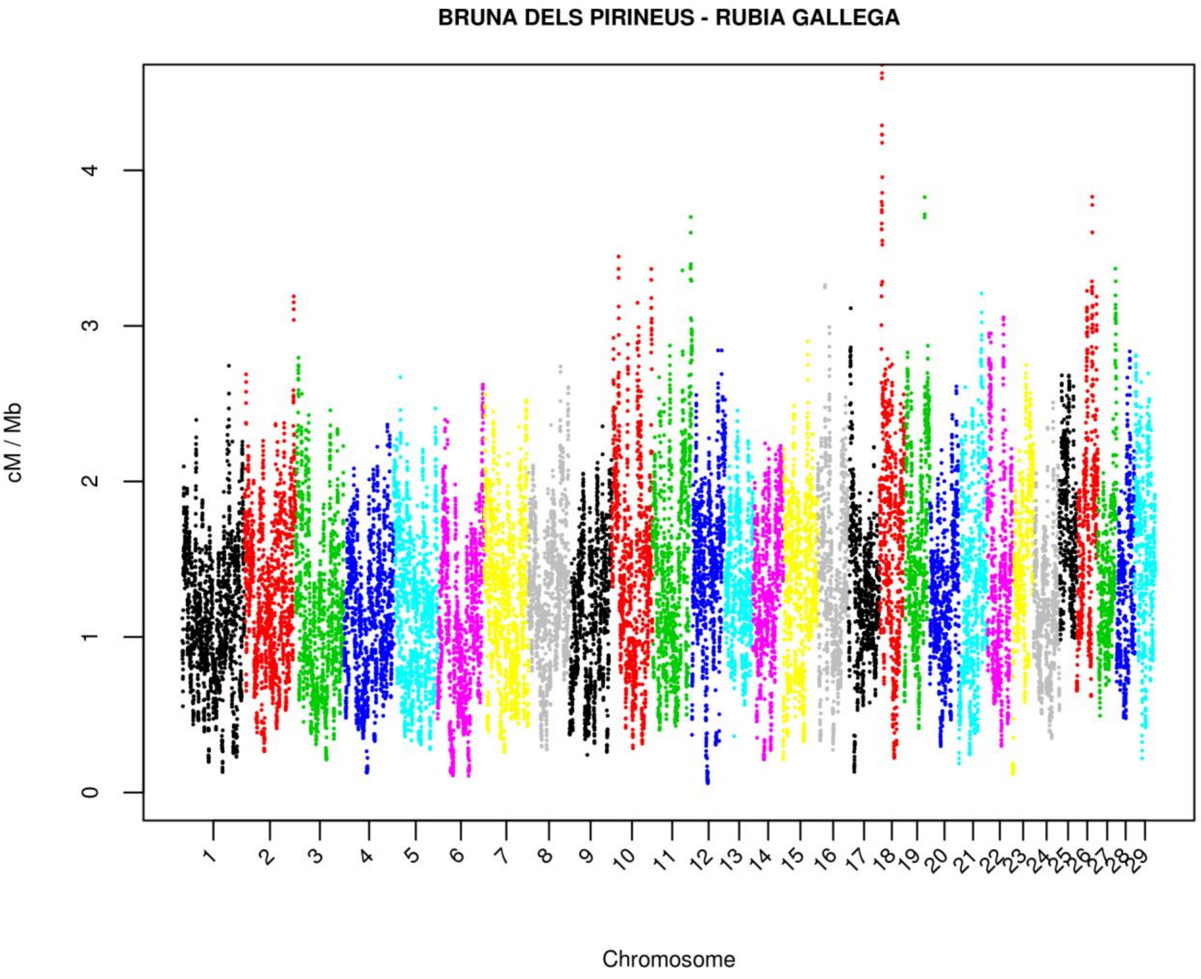

Supplementary Figure 36. *Estimates of the recombination rate (c) in bins of 1 Mb between Morucha -Mo- and Pirenaica -Pi- throughout the autosomal genome.*

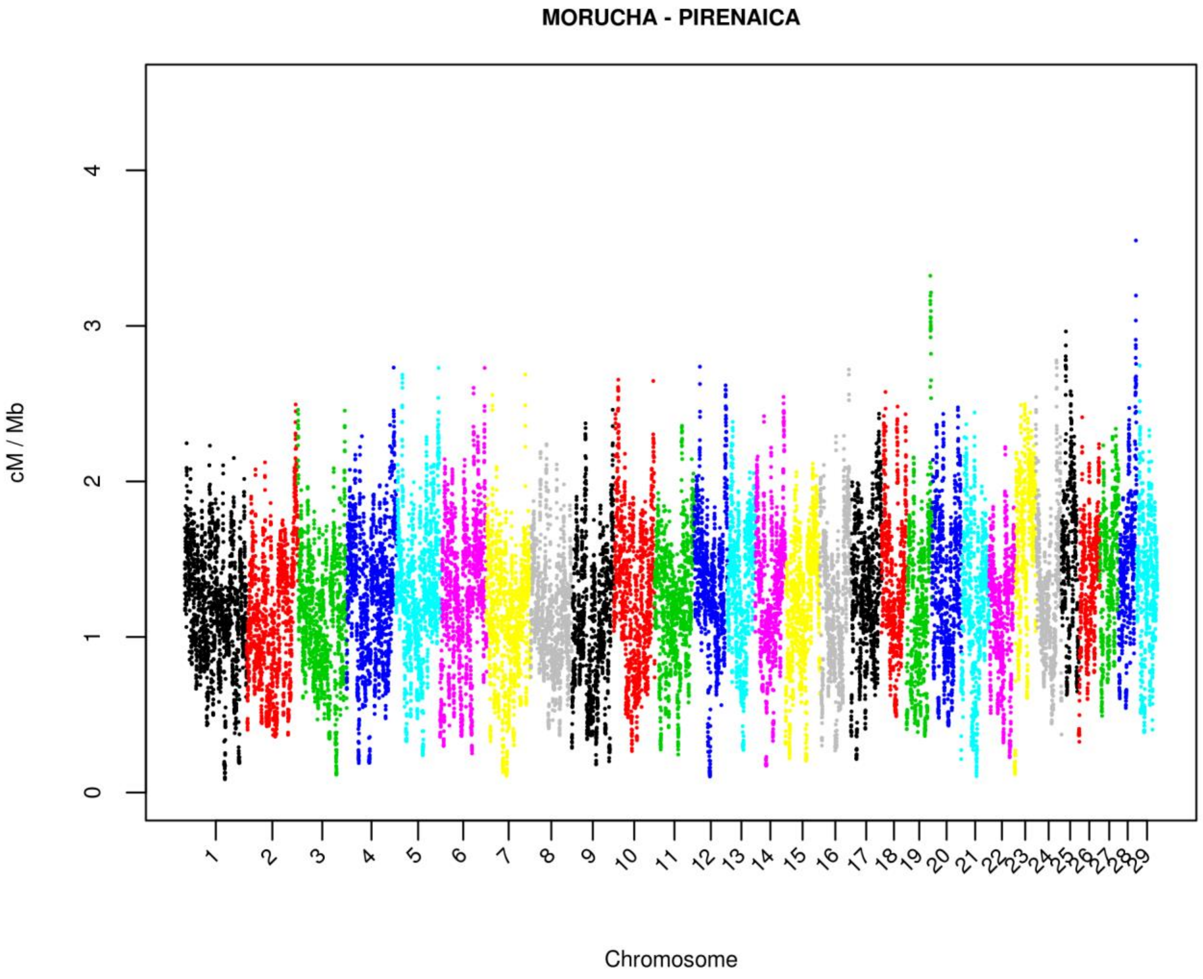

Supplementary Figure 37. *Estimates of the recombination rate (c) in bins of 1 Mb between Morucha -Mo- and Retinta -Re- throughout the autosomal genome.*

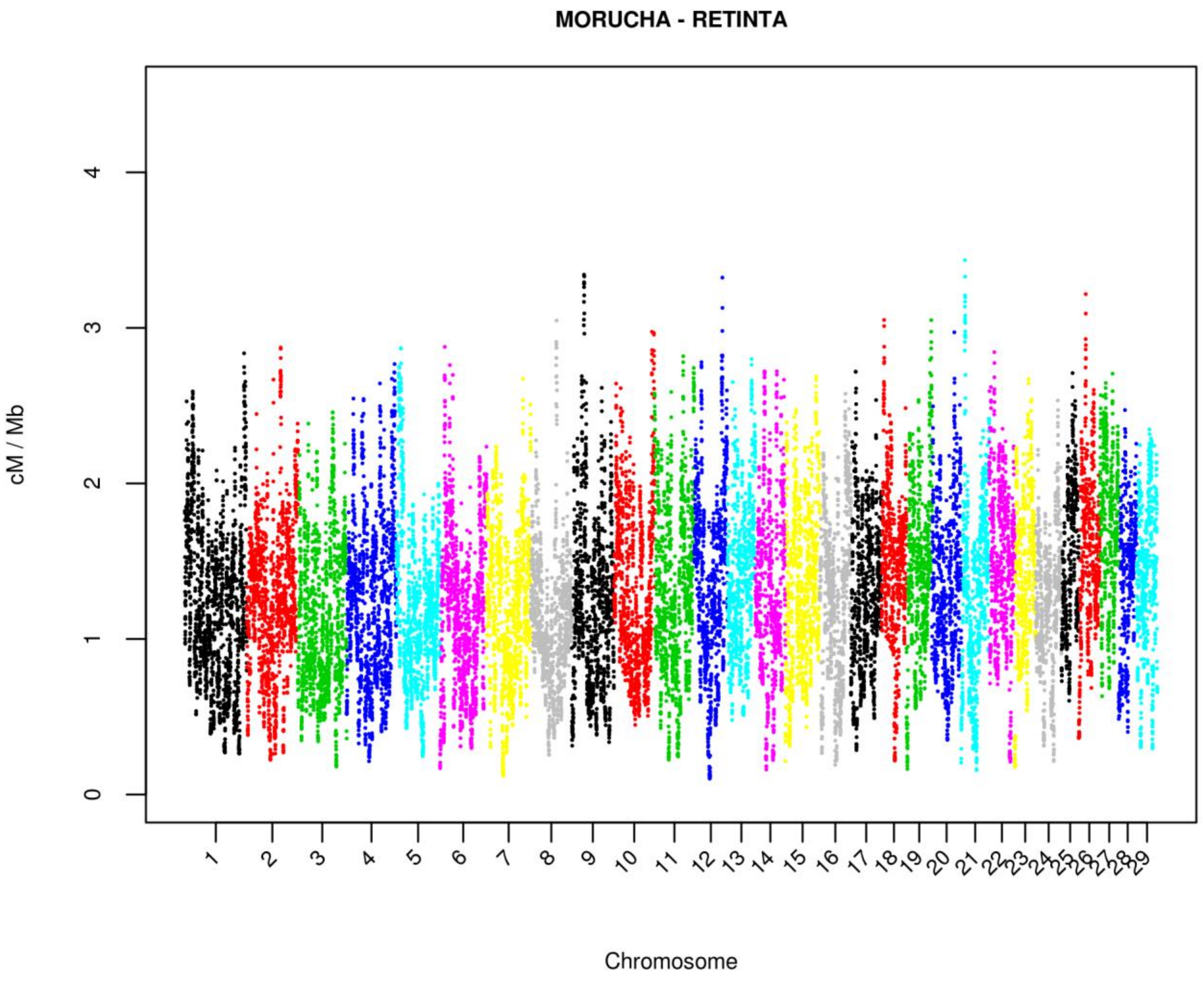

Supplementary Figure 38. *Estimates of the recombination rate (c) in bins of 1 Mb between Morucha -Mo- and Rubia Gallega -RG- throughout the autosomal genome.*

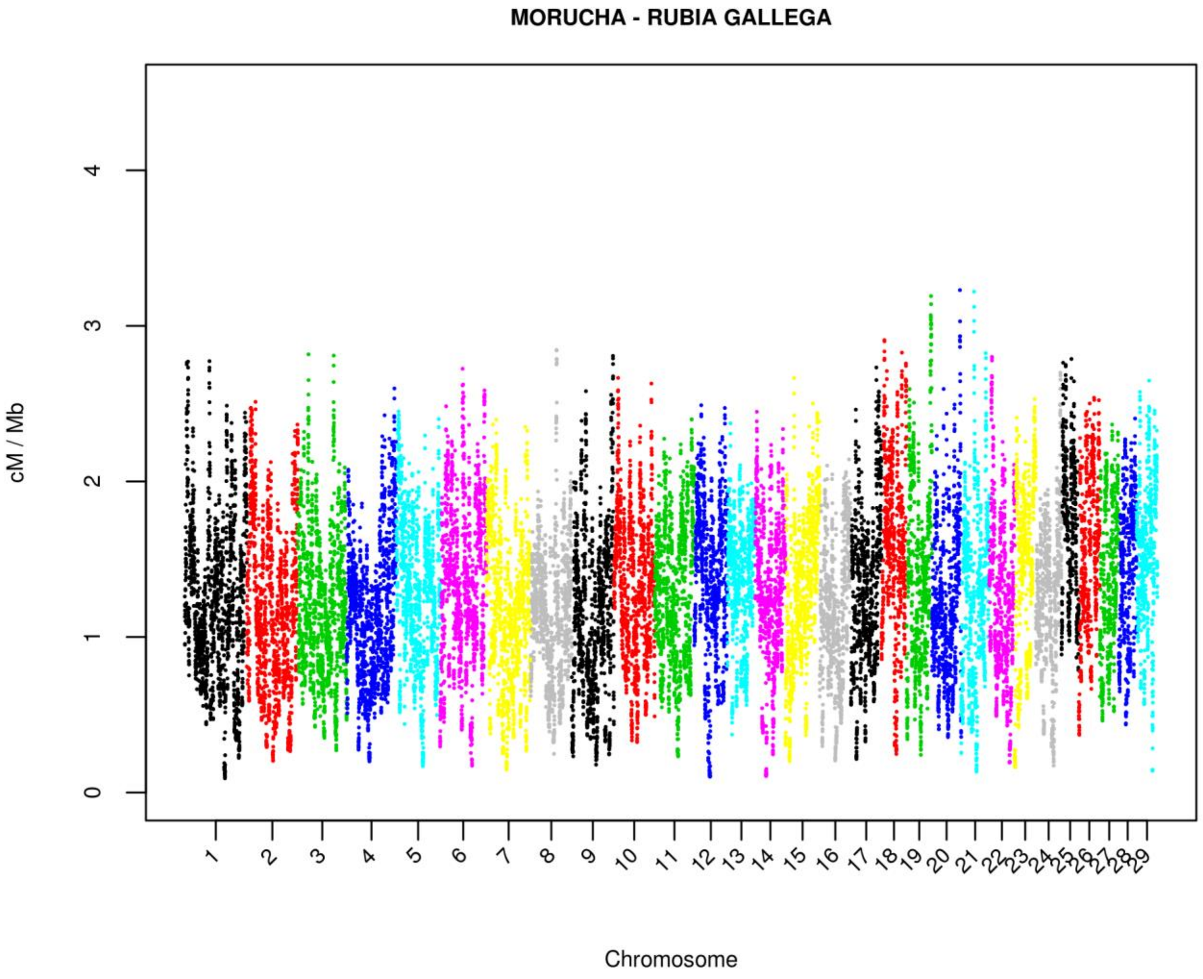

Supplementary Figure 39. *Estimates of the recombination rate (c) in bins of 1 Mb between Pirenaica -Pi- and Retinta -Re- throughout the autosomal genome.*

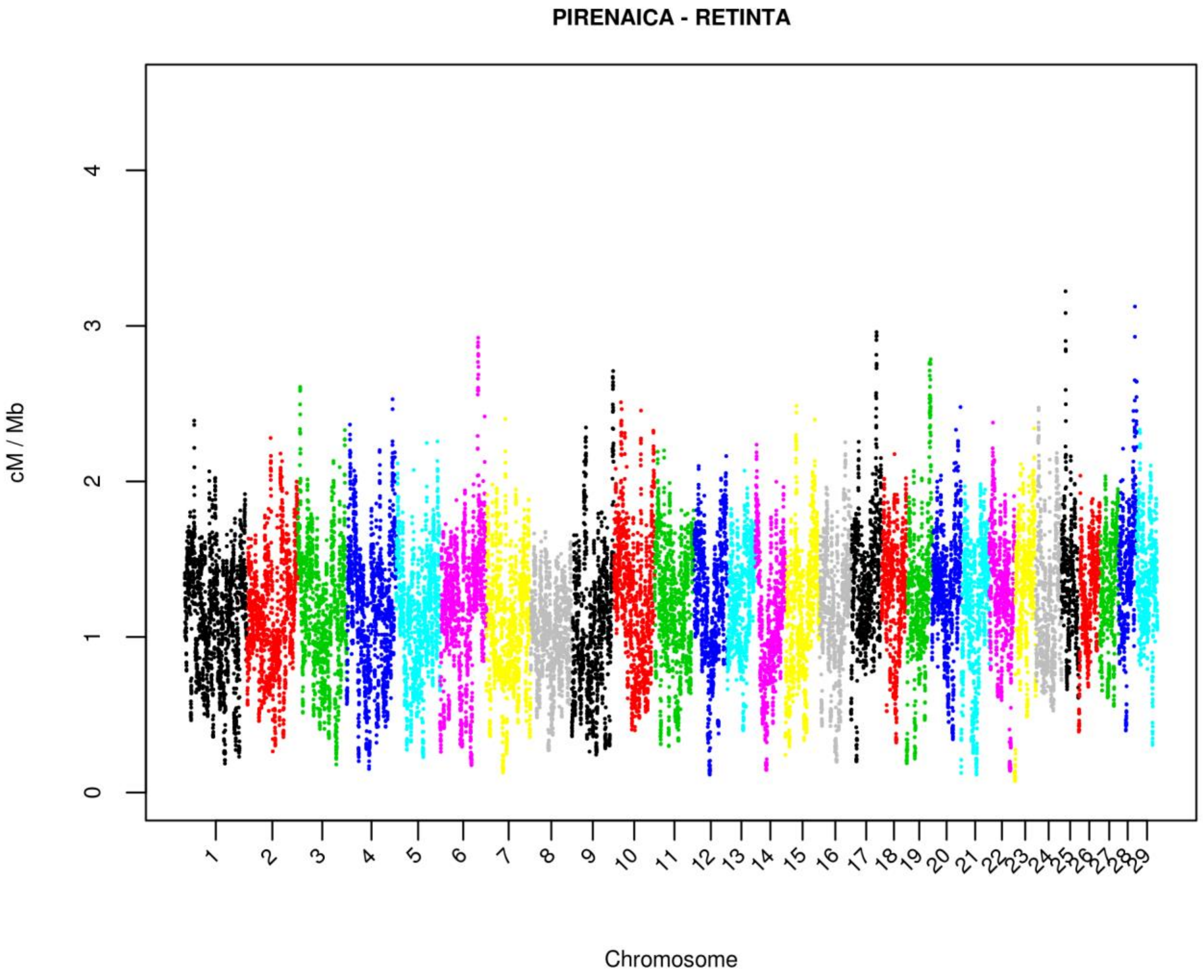

Supplementary Figure 40. *Estimates of the recombination rate (c) in bins of 1 Mb between Pirenaica -Pi- and Rubia Gallega -RG- throughout the autosomal genome.*

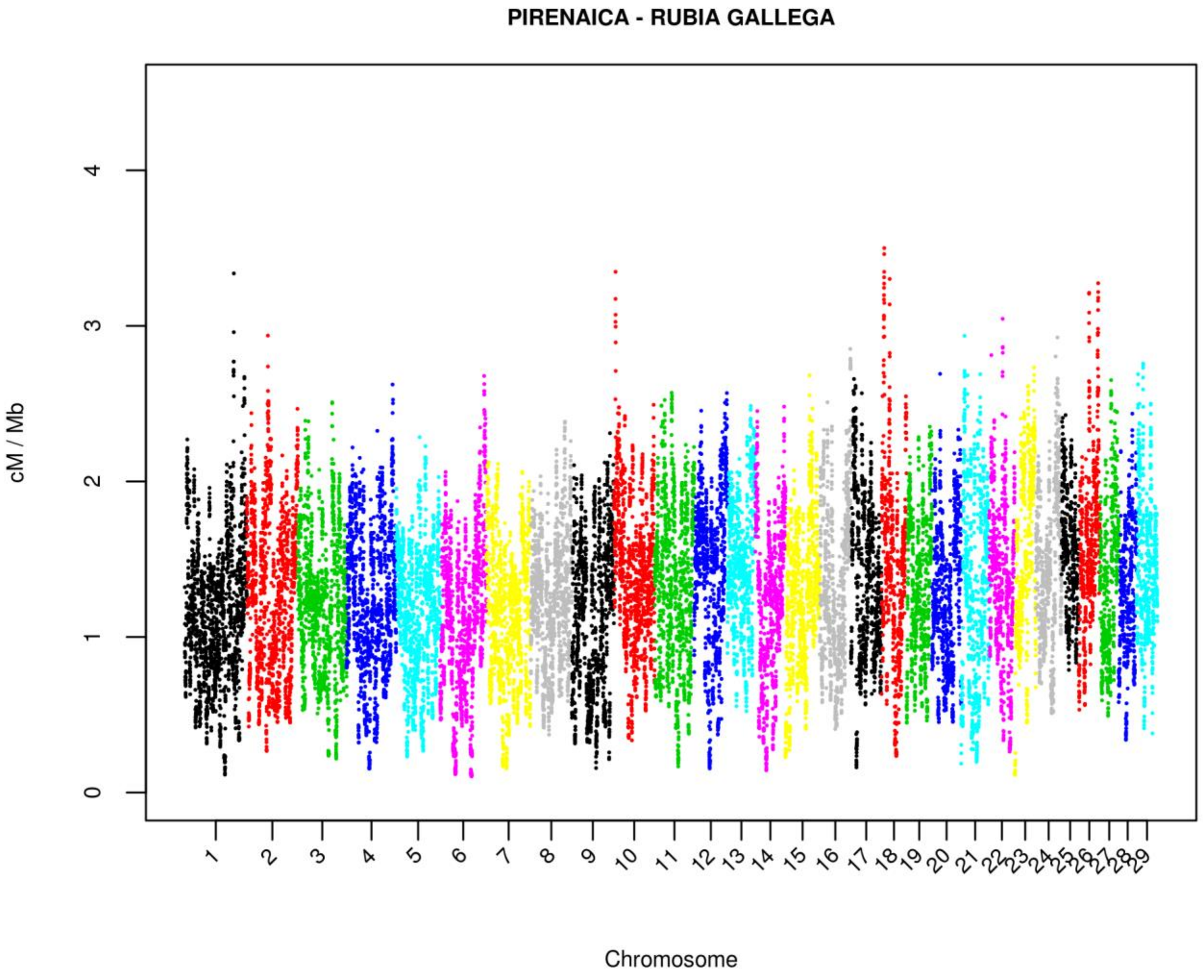

Supplementary Figure 41. *Estimates of the recombination rate (c) in bins of 1 Mb between Retinta -Re- and Rubia Gallega -RG- throughout the autosomal genome.*

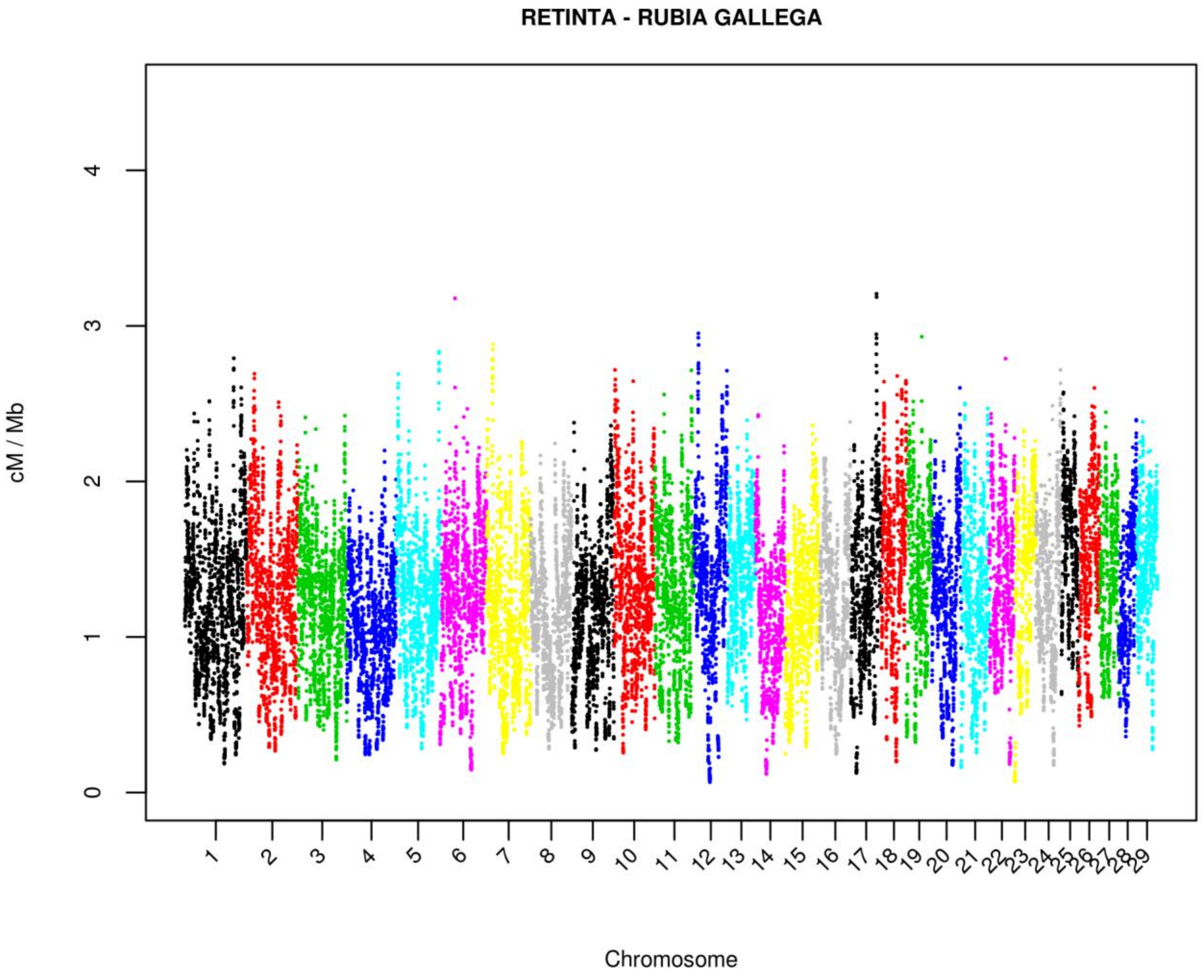

Supplement: Supplementary file 1 [file DataSheet_1.pdf]
